# Scalable Lignin Monomer Production Via Machine Learning‐Guided Reductive Catalytic Fractionation of Lignocellulose

**DOI:** 10.1002/advs.202510496

**Published:** 2025-08-27

**Authors:** Meysam Madadi, Ehsan Kargaran, Seyed Sajad Hashemi, Chihe Sun, Joeri F.M. Denayer, Keikhosro Karimi, Fubao Sun, Vijai Kumar Gupta

**Affiliations:** ^1^ Key Laboratory of Industrial Biotechnology Ministry of Education School of Biotechnology Jiangnan University Wuxi 214122 China; ^2^ Department of Chemical Engineering Vrije Universiteit Brussel Brussels 1050 Belgium; ^3^ School of Biotechnology Dublin City University Glasnevin Dublin D09 K20V Ireland

**Keywords:** biomass, catalytic fractionation, lignin, machine learning, sustainable scaling

## Abstract

Efficient valorization of lignocellulosic biomass into high‐value lignin monomers is a cornerstone of sustainable biorefineries, yet the complexity of optimizing reductive catalytic fractionation limits industrial scalability. This study presents a machine learning (ML)‐driven framework that harnesses 3,451 experimental data points from 54 peer‐reviewed studies to model and optimize lignin monomer production. Among four advanced ML models developed, eXtreme Gradient Boosting Regression is found to achieve the highest predictive accuracy (R = 0.80–0.86) with low prediction errors (root mean square error: 3.99–8.31; mean absolute error: 2.85–6.90) for monomer production. Feature importance analysis reveals that operational parameters account for the largest influence (40–57%), followed by substrate content (25–43%) and catalyst‐solvent properties (14–21%). The error between experimental and ML‐predicted total monomer yields ranges from 2% to 2.6%, demonstrating robust performance of the model. Scaling this approach has the potential to process 140 million tons of aspen biomass annually, can reduce CO_2_ emissions by 20.6 million tons, and yield $4,729 million in socioeconomic savings. This ML‐enhanced strategy offers a scalable and environmentally viable pathway for data‐driven lignocellulose valorization, advancing the development of low‐carbon, economically competitive biorefineries.

## Graphical abstract

This study pioneers an integrated machine learning framework combining large‐scale data modeling, experimental validation, and impact analysis to optimize reductive catalytic fractionation for scalable, sustainable lignin monomer production.

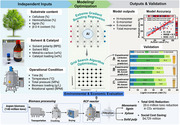

## 1 Introduction

The transition to a sustainable, circular bioeconomy hinges on the efficient valorization of lignocellulosic biomass (LCB), a renewable and abundant resource. LCB, composed of cellulose, hemicellulose, and lignin, offers a unique opportunity to produce renewable fuels, chemicals, and materials to reduce reliance on fossil resources.^[^ ^1^ ^]^ Among its components, lignin—for example, the complex aromatic polymer—offers great potential as a feedstock for producing high‐value industrial monomeric phenols, such as guaiacol and syringol, which are widely used in fuels, resins, and fine chemicals.^[^ ^2^, ^3^ ^]^ However, the inherent resistance of lignin to depolymerization, driven by multiple interacting factors such as substrate composition, solvent polarity, catalyst properties, and reaction conditions, has historically hindered its utilization; as a result, most lignin has been combusted for low‐value energy recovery.^[^ ^4^ ^]^

Reductive catalytic fractionation (RCF) emerges as a promising “lignin‐first” biorefinery strategy that offers an efficient approach to valorizing LCB. This process facilitates the selective extraction of lignin‐derived products while preserving polysaccharides in a largely unaltered state.^[^ ^5^ ^]^ Among various lignin valorization strategies, RCF offers distinct advantages due to its integrated approach to lignin‐first processing.^[^ ^2^ ^]^ Unlike traditional thermochemical or oxidative depolymerization methods, which often result in low selectivity and condensed byproducts, RCF selectively cleaves labile ether linkages—particularly β‐O‐4 bonds—under reductive conditions that suppress recondensation.^[^ ^6^ ^]^ This enables high yields of stable, low‐molecular‐weight monomeric phenols. Additionally, RCF is typically performed in the presence of metal catalysts and hydrogen donors or reductive solvents, which not only enhance depolymerization efficiency but also stabilize reactive intermediates.^[^ ^7^ ^]^ The simultaneous preservation of cellulose‐rich pulp further positions RCF as a promising pathway for integrated biorefinery applications, combining high lignin monomer recovery with carbohydrate valorization.^[^ ^8^ ^]^

The RCF process generally involves three key steps: i) solvolytic delignification to isolate lignin from LCB, ii) catalytic and solvolytic depolymerization of lignin into smaller fragments, and iii) stabilization of the resulting lignin monomers to prevent repolymerization/condensation.^[^ ^9^, ^10^ ^]^ A defining feature of RCF is the use of a heterogeneous redox catalyst, which, in the presence of hydrogen gas (H_2_) or hydrogen donors, enables both lignin depolymerization and the stabilization of reactive intermediates.^[^ ^11^ ^]^ This process primarily yields low molecular weight lignin monomers, alongside some dimers and oligomers. The monomeric yields (*p*‐hydroxyphenyl (H), guaiacyl (G), syringyl (S), and total monomer yields) are highly dependent on several variables, including the source of LCB (e.g., woody biomass and herbaceous crops), the choice of solvent (e.g., ethanol and methanol), and catalyst (e.g., Ru, Pd, and Rh), and operational conditions (e.g., reaction time, temperature, total pressure, biomass loading, and rotation speed).^[^ ^6^, ^12^, ^13^, ^14^ ^]^

The composition and structure of lignin vary significantly among different LCB sources, which directly impacts monomer yields. Hardwood lignin, characterized by a higher β‐O‐4 bond content and a greater proportion of S units, generally produces higher monomer yields (31.9–46.7%) compared to softwood lignin (12.9–15.5%) and herbaceous biomass lignin (20.0–46.0%).^[^ ^15^ ^]^ Despite herbaceous biomass lignin possessing the highest β‐O‐4 linkage content (74.0–84.0%), its monomer yields are often limited by high ash content, which deactivates catalysts, and by hydroxycinnamic acids such as ferulic and p‐coumaric acids, which promote lignin repolymerization through side reactions.^[^ ^6^ ^]^ Similarly, bark lignin, due to its more condensed structure, generates lower monomer yields than wood lignin.^[^ ^6^, ^16^ ^]^ The choice of solvent also plays a critical role in determining lignin monomer yields. Solvent properties such as polarity and solvent relative energy difference (RED) significantly influence lignin extraction and depolymerization efficiency.^[^ ^17^ ^]^ Polar protic solvents, such as methanol and ethanol, are particularly effective in dissolving lignin and cleaving β‐O‐4 bonds. Methanol often outperforms ethanol, with studies reporting phenolic monomer yields of up to 51.4% using 60% aqueous methanol. Hydrogen‐donor solvents like isopropanol enhance selectivity for specific alkyl phenols. Low‐vapor solvents, such as 1,2‐propanediol, achieve comparable yields without external hydrogen, improving process efficiency.^[^ ^6^, ^12^ ^]^

Catalysts, particularly bifunctional ones that combine metal sites with acidic supports, are another critical factor. The metal facilitates hydrogenation, while the acidic sites promote C─O bond cleavage. For example, Ru‐based catalysts on activated carbon (Ru/C) have demonstrated high monomer yields (up to 57.98%) due to superior metal dispersion and hydrogenation capability. Similarly, zeolite‐based catalysts like HZSM‐5 enhance depolymerization while suppressing recondensation. Catalyst properties, including metal‐to‐carbon ratio and loading dosage, significantly influence both lignin depolymerization efficiency and phenolic monomer selectivity.^[^ ^6^, ^13^, ^18^ ^]^ Operational conditions further dictate monomer yields. Longer reaction times generally improve delignification but may also lead to monomer recondensation, reducing overall yield. Elevated temperatures and pressures enhance lignin depolymerization but can trigger undesirable side reactions if not carefully controlled. Optimal biomass loading ensures effective interaction with the catalyst, whereas excessive loading hinders mass transfer and reduces yields. Similarly, higher mixing speeds improve mass transfer, enhancing monomer production.^[^ ^12^, ^13^, ^19^ ^]^ Despite significant advancements, optimizing lignin monomer yields remains challenging due to the intricate interplay of these parameters. Defining a universally accepted set of optimal conditions is particularly difficult, necessitating further research and fine‐tuning to achieve efficient lignin valorization.

Machine learning (ML) offers a powerful solution to address these challenges by leveraging large experimental datasets to model complex relationships and predict outcomes.^[^ ^20^, ^21^ ^]^ ML provides key advantages, including reduced experimental costs, faster optimization processes, and strong generalization capabilities.^[^ ^22^ ^]^ It has already proven effective in designing solvents and catalysts, optimizing reaction conditions, and evaluating the impact of operational parameters on LCB fractionation and lignin upgrading.^[^ ^21^ ^]^ For instance, Lee et al.^[^ ^23^ ^]^ developed an ML‐based framework to optimize catalytic pretreatment conditions for oil palm mesocarp fibers, achieving $1.45 billion in annual profit with operating costs of $40 per ton. Mattern et al.^[^ ^24^ ^]^ employed ML to design novel solvents for LCB fractionation, identifying high‐performing solvents such as azoles and glycol ethers with high lignin solubility (20–60%), low toxicity, and acid stability. Toyao et al.^[^ ^25^ ^]^ highlighted the potential of ML to accelerate catalyst discovery and improve understanding of material properties, though its application in catalyst development remains limited due to the complexity of catalytic processes. However, research integrating ML with RCF remains limited. Existing studies have primarily focused on basic ML techniques, such as PCA, to identify correlations between LCB composition and monomer yields.^[^ ^26^ ^]^ To address this gap, this research presents an ML‐driven framework to overcome the limitations and challenges identified in existing studies.

This study integrates ML and experimental validation to optimize RCF for lignin monomer production. A systematic literature review compiles data on LCB composition, catalyst‐solvent properties, and operational parameters, which are analyzed using statistical and mechanistic approaches. Four ML models are developed, with the best‐performing model used for feature importance analysis to identify key variables influencing monomer yields. Optimized conditions are experimentally validated for accuracy. The study also evaluates environmental impacts and socioeconomic benefits, demonstrating the feasibility of scaling this approach for sustainable biorefineries in China and beyond.

## 2 Results and Discussion

### 2.1 Statistical Data Analysis of Modeling Data

**Figure 1:**
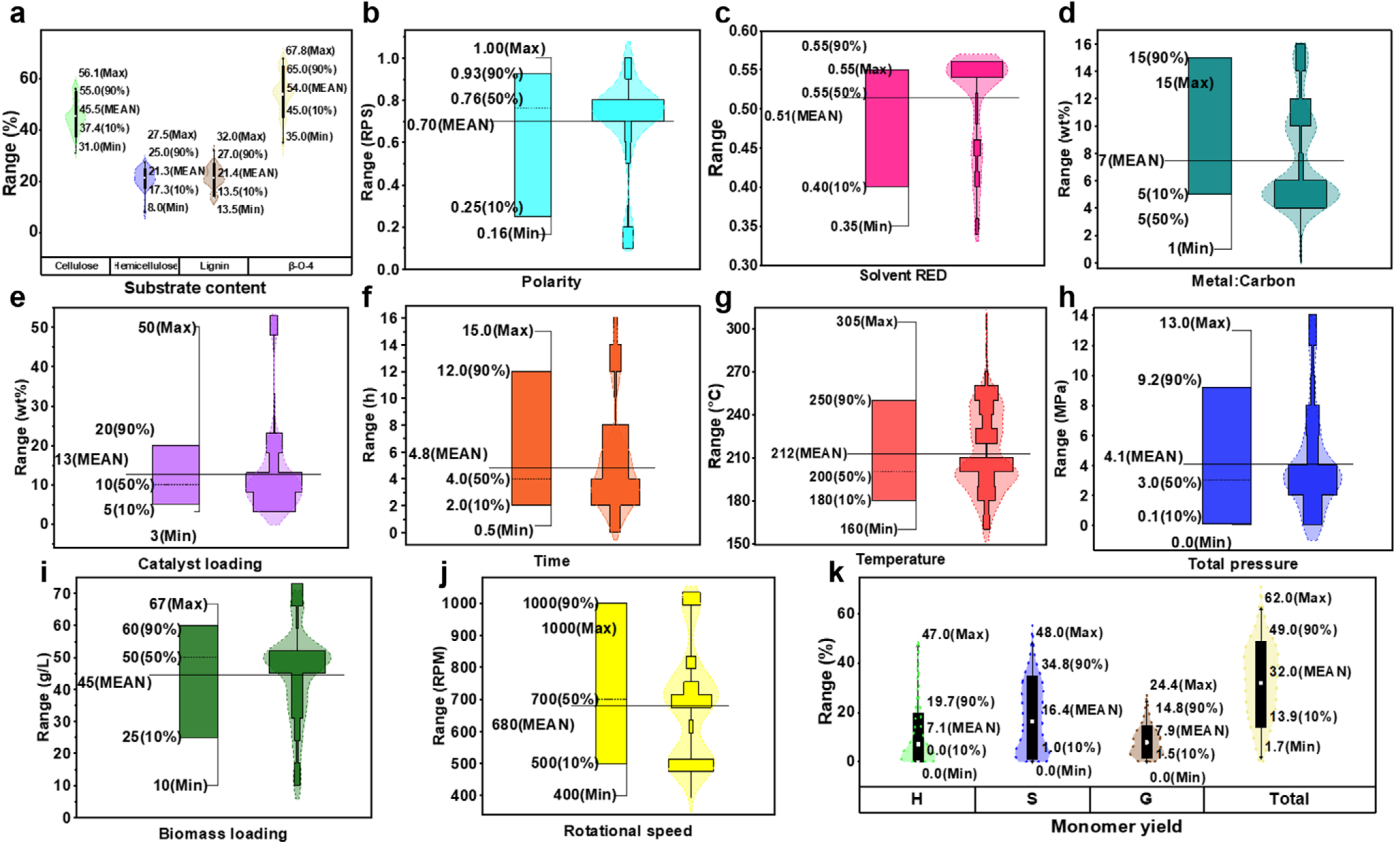
Input and output variables used in developing ML models. Substrate content a), solvent polarity b), solvent RED c), metal‐to‐carbon ratio d), catalyst loading e), time f), temperature g), total pressure h), biomass loading i), rotational speed j), and monomer yields k).

The detailed statistical evaluation of the database was used to identify trends and analyze the factors influencing monomeric lignin production through RCF. **Figure** **1** showcases important statistical measures, including the maximum (max), mean, and minimum (min) of all inputs and output variables.

The analysis revealed significant variability in LCB content, with cellulose content ranging from 31.0% to 56.1% (mean: 45.5%), hemicellulose from 8.0% to 27.5% (mean: 17.8%), and lignin from 13.5% to 32.0% (mean: 22.7%). Notably, the β‐O‐4 content, a key structural determinant of monomer yields,^[^ ^16^ ^]^ ranged from 34.0% to 67.8%, with an average of 54% (Figure 1a). Solvent properties exhibited moderate variability, with solvent polarity ranging from 0.16 to 1 RPS (mean: 0.7 RPS) and relative energy difference (RED) values spanning 0.35–0.55 (mean: 0.51) (Figure 1b,c). Catalyst‐related parameters displayed considerable diversity, with metal‐to‐carbon ratios ranging from 1 to 15 wt.% and catalyst loadings spanning 3–50 wt.% (Figure 1d,e). These variations reflect the broad spectrum of experimental setups employed in RCF studies, highlighting the complexity of optimizing catalyst systems. Operational conditions demonstrated substantial variability. Reaction temperatures ranged from 160 to 305 °C (mean: 212 °C), reaction times from 0.5 to 15 h (mean: 4.8 h), and total pressure from 0 to 13 MPa (mean: 4.1 MPa). Biomass loadings spanned 10–67 g L^−1^, while rotational speeds varied between 400 and 1000 RPM (Figure 1f–j). This wide range of parameters emphasizes the flexibility of RCF processes and the challenges associated with standardizing optimal conditions. The output data revealed that S‐monomer yields were the largest contributors to total monomer yields, ranging from 0% to 48% (mean: 16.4%), followed by H‐monomer yields (0–47%, mean: 7.1%) and G‐monomer yields (0–24.4%, mean: 7.9%). Total monomer yields ranged from 34% to 67.8%, with an average of 54%. In the monomer distribution, 90% of the data reported S‐monomers at 34.8%, H‐monomers at 19.7%, and G‐monomers at 14.8% (Figure 1k), reflecting the prevalence of S‐type lignin in most LCB feedstocks.

The variability in input variables highlights the multifactorial complexity of the RCF process. This diversity provides a comprehensive dataset, avoiding excessive clustering or dispersion. As such, the dataset serves as a robust foundation for developing advanced ML models capable of generalizing across diverse experimental conditions. These models have the potential to optimize monomeric lignin production and deepen the understanding of the RCF process, paving the way for more efficient LCB valorization strategies.

**Figure 2:**
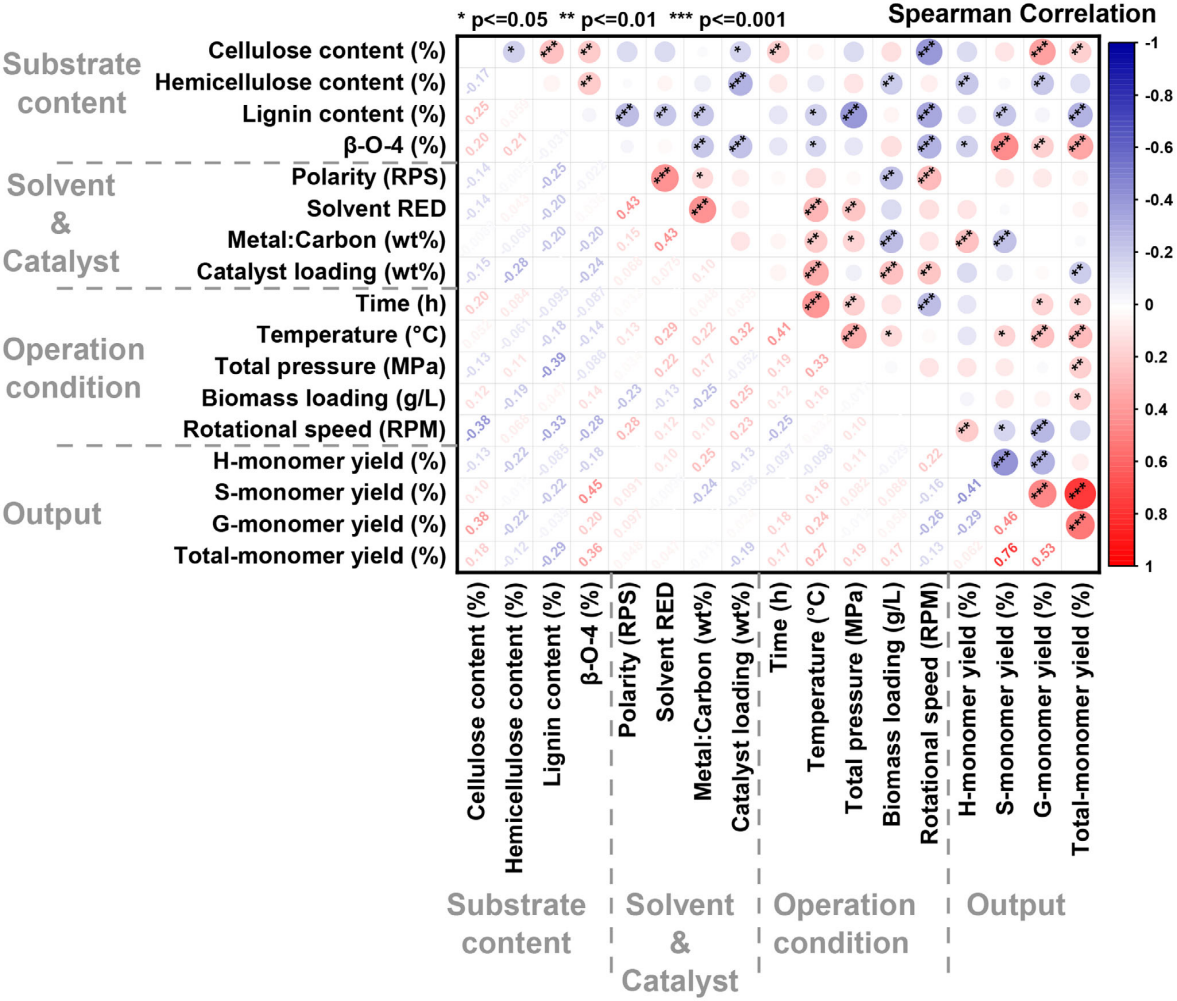
Heatmap displaying Spearman correlation coefficients and their corresponding *p*‐values between input and output variables.

The Spearman rank correlation method is a non‐parametric approach used to evaluate the strength and direction of relationships between variables. It calculates similarity based on data ranks, with a coefficient of +1 indicating a perfect positive correlation and −1 representing a complete negative correlation. In a correlation matrix heatmap, the diagonal values are always +1, as each variable is perfectly correlated with itself.^[^ ^27^ ^]^ This method is particularly useful for identifying dependencies and associations, such as in monomeric lignin production via RCF. **Figure** **2** shows the Spearman correlation matrix, highlighting the relationships between input variables and output targets in the dataset.

The analysis revealed that cellulose content showed a strong positive correlation with G‐monomer yield (r = 0.38, *p* ≤ 0.001) and a weak positive correlation with total monomer yield (r = 0.18, *p* ≤ 0.01). Conversely, hemicellulose content exhibited a moderate negative correlation with both H‐monomer yield (r = −0.22, *p* ≤ 0.01) and G‐monomer yield (r = −0.22, *p* ≤ 0.01), suggesting that hemicellulose content hinders the production of these monomers. Lignin content displayed a moderate negative correlation with S‐monomer yield (r = −0.22, *p* ≤ 0.01) and a strong negative correlation with total monomer yield (r = −0.29, *p* ≤ 0.001), indicating that higher lignin content reduces both S‐monomer and overall monomer production.

β‐O‐4 content, a key structural linkage in lignin, showed a strong positive correlation with S‐monomer yield (r = 0.45, *p* ≤ 0.001), a weak positive correlation with G‐monomer yield (r = 0.20, *p* ≤ 0.01), and a moderate positive correlation with total monomer yield (r = 0.36, *p* ≤ 0.001). However, it exhibited a weak negative correlation with H‐monomer yield (r = ‐0.18, *p* ≤ 0.05). These results highlight the importance of β‐O‐4 linkages in facilitating the depolymerization of lignin to produce S‐ and G‐monomers and increase overall monomer yield.^[^ ^13^, ^16^ ^]^ No significant correlations were observed between solvent properties and monomer yields, indicating that solvent characteristics do not significantly influence monomer production under the studied conditions. The metal‐to‐carbon ratio of the catalyst demonstrated a strong positive correlation with H‐monomer yield and a strong negative correlation with S‐monomer yield, indicating opposing effects on these monomer types. In contrast, catalyst loading showed a weak negative correlation with total monomer yield (r = −0.19, *p* ≤ 0.01), suggesting that higher catalyst loading slightly reduces overall monomer production.

Among the operational factors, all conditions except rotational speed exhibited moderate to strong positive correlations with total monomer yield, with correlation coefficients ranging from 0.17 to 0.27. Notably, temperature showed a moderate positive correlation with S‐monomer yield (r = 0.16, *p* ≤ 0.01) and strong positive correlations with G‐monomer yield (r = 0.24, *p* ≤ 0.001) and total monomer yield (r = 0.27, *p* ≤ 0.001). These findings underscore the critical role of temperature in enhancing S‐ and G‐monomer yields and improving overall lignin depolymerization efficiency.^[^ ^6^ ^]^ In summary, the reported studies highlight the pivotal role of β‐O‐4 content and temperature in enhancing S‐ and G‐monomer production, as well as improving the total monomer yield.

**Figure 3:**
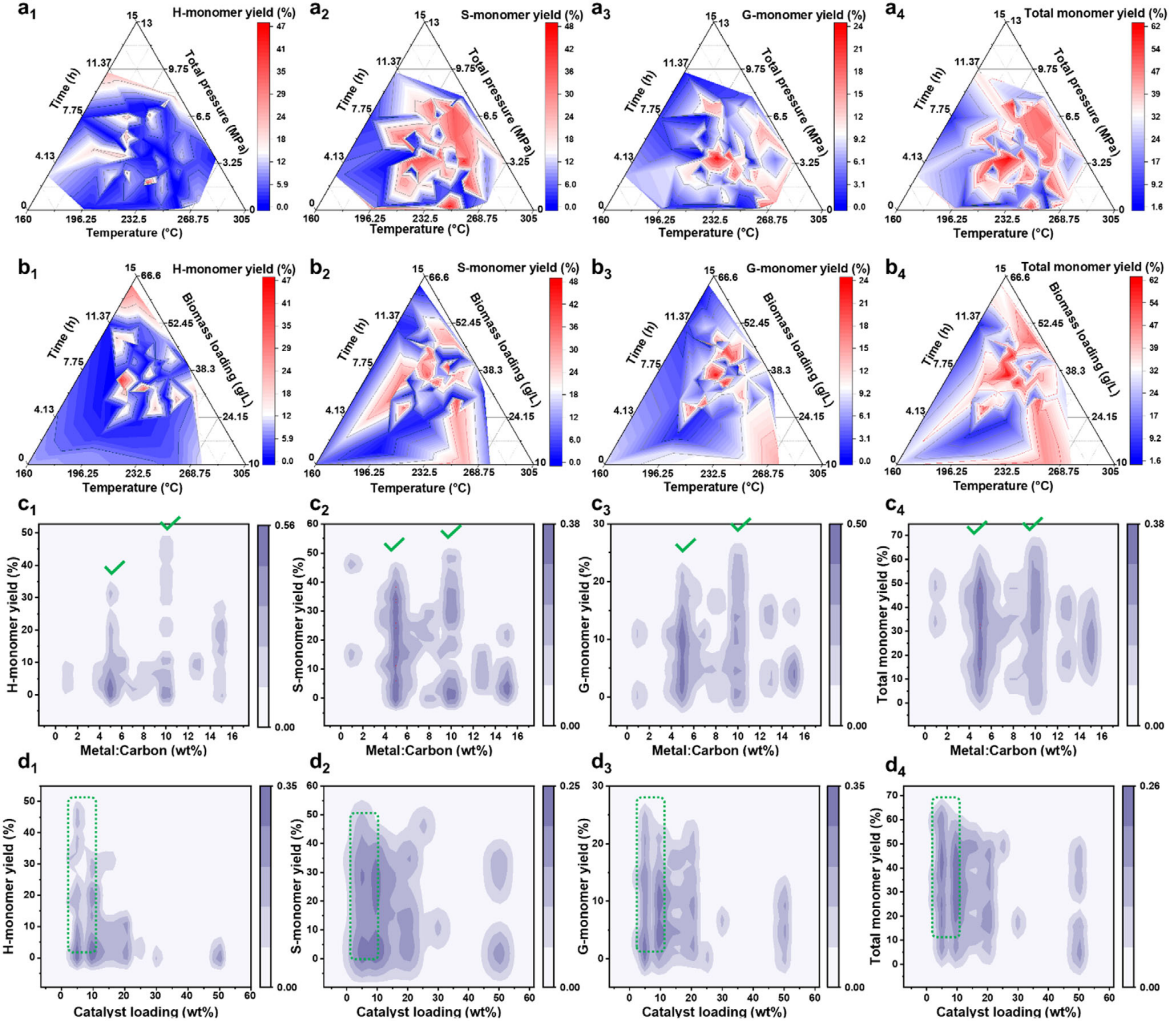
Effects of key operational conditions (time, temperature, total pressure, and biomass loading) on the yields of a_1_,b_1_) H‐, a_2_,b_2_) S‐, a_3_,b_3_) G‐, and a_4_,b_4_) total monomer yields. Kernel density distribution of metal‐to‐carbon ratio and catalyst loading on c_1_,d_1_) H‐, c_2_,d_2_) S‐, c_3_,d_3_) G‐, and c_4_,d_4_) total monomers yields.

As indicated by the correlation analysis, operational conditions (excluding rotational speed) showed positive correlations with total monomer yield. Moreover, numerous review papers have emphasized a strong relationship between these variables.^[^ ^6^, ^12^, ^13^, ^14^ ^]^ To gain deeper insights, a ternary analysis was conducted to elucidate the relationship between operational conditions and monomer yields (**Figure** **3**a,b). Higher H‐monomer yields are achieved when the temperature, time, total pressure, and biomass loading range between 175 and 195 °C, 8–10 h, 1.5–2 MPa, and 30–35 g L^−1^, respectively. Notably, increasing biomass loading, along with longer times and higher total pressures at lower temperatures, enhances H‐monomer production. Temperature appears to have minimal impact, while the relationship with total pressure remains ambiguous (Figure 3a_1_,b_1_). For S‐monomer, time has the lowest impact, while moderate biomass loadings (≈25–40 g L^−1^) improve production, but both higher and lower loadings reduce yields. The effect of total pressure remains unclear; however, increasing the temperature within the range of 180–245 °C promotes S‐monomer production (Figure 3a_2_,b_2_). Higher G‐monomer yields are obtained when the temperature, reaction time, total pressure, and biomass loading range between 195 and 220 °C, 10–12 h, 2–4 MPa, and 30–45 g L^−1^, respectively. Moderate pressures (≈2–4 MPa) enhance yield, while biomass loading and reaction time have minimal effects. Increasing the temperature up to 220 °C promotes production, but higher temperatures negatively affect S‐monomer yields (Figure 3a_3_,b_3_). For total monomer yields, optimal conditions occur when the temperature, reaction time, total pressure, and biomass loading range between 185 and 235 °C, 5–12 h, 1–3.5 MPa, and 30–45 g L^−1^, respectively. Moderate pressures (≈2–4 MPa) enhance yields, while biomass loading and reaction time have significant effects. Moderate to higher temperatures (≈180–235 °C) promote production, but higher temperatures reduce total monomer yields (Figure 3a_4_,b_4_).

To better understand the varying impact of catalyst properties on monomer yields, a Kernel density distribution of the metal‐to‐carbon ratio and catalyst loading was analyzed. The analysis revealed that a metal‐to‐carbon ratio of 5 wt.%, and particularly 10 wt.%, produced the highest monomer yields (Figure 3c_1_–c_4_). While increased metal loading provides more active sites for the reaction, excessive amounts can lead to particle aggregation, reducing catalytic efficiency.^[^ ^28^, ^29^ ^]^ Similarly, catalyst loadings in the range of 5–10 wt.% consistently achieved the highest yields (Figure 3d_1_–d_4_). Although higher catalyst loadings can improve lignin conversion rates, excessive amounts may result in diminishing returns and complicate comparisons across studies.^[^ ^30^ ^]^

Principal Component Analysis (PCA) is a widely used statistical technique in data analysis, designed to identify hidden patterns and relationships within datasets. It is particularly effective for reducing dimensionality and identifying the most significant components that drive overall variance.^[^ ^31^ ^]^ In the context of monomer production from the RCF dataset, PCA is employed to analyze and interpret the complex interactions between variables. The first three principal components (PCs) for H‐ (Figure S1a_1_, Supporting Information), S‐ (Figure S1a_2_, Supporting Information), G‐ (Figure S1a_3_, Supporting Information), and total monomer yields (Figure S1a_4_, Supporting Information) accounted for 44.7%, 46.5%, 45.7%, and 46.6% of the total variance, respectively. Across all cases (H‐, S‐, G‐, and total monomer yields), PC1 was primarily associated with variables such as rotational speed, metal‐to‐carbon ratio, solvent RED, lignin content, and cellulose content. In contrast, PC2 and PC3 exhibited strong correlations with time, temperature, and β‐O‐4 content, and moderate correlations with catalyst loading, hemicellulose, and biomass loading. Based on this analysis, operational conditions (particularly time and temperature) and substrate content (particularly β‐O‐4 and lignin content) accounted for the major input variables impacting the monomer yields (Figure S1(b_1_–b_4_), Supporting Information). It should be noted that the first three PCs were unable to capture a significant portion of the dataset's variability, highlighting the inherent complexity of the RCF process. This complexity arises from multifactorial interactions and the substantial influence of numerous variables on the output targets, which cannot be fully explained by PCA alone. To address these limitations, advanced ML techniques such as SHAP offer a powerful approach to uncovering deeper and more nuanced insights into these intricate relationships.

### 2.2 Modeling and Feature Importance Results

**Figure 4:**
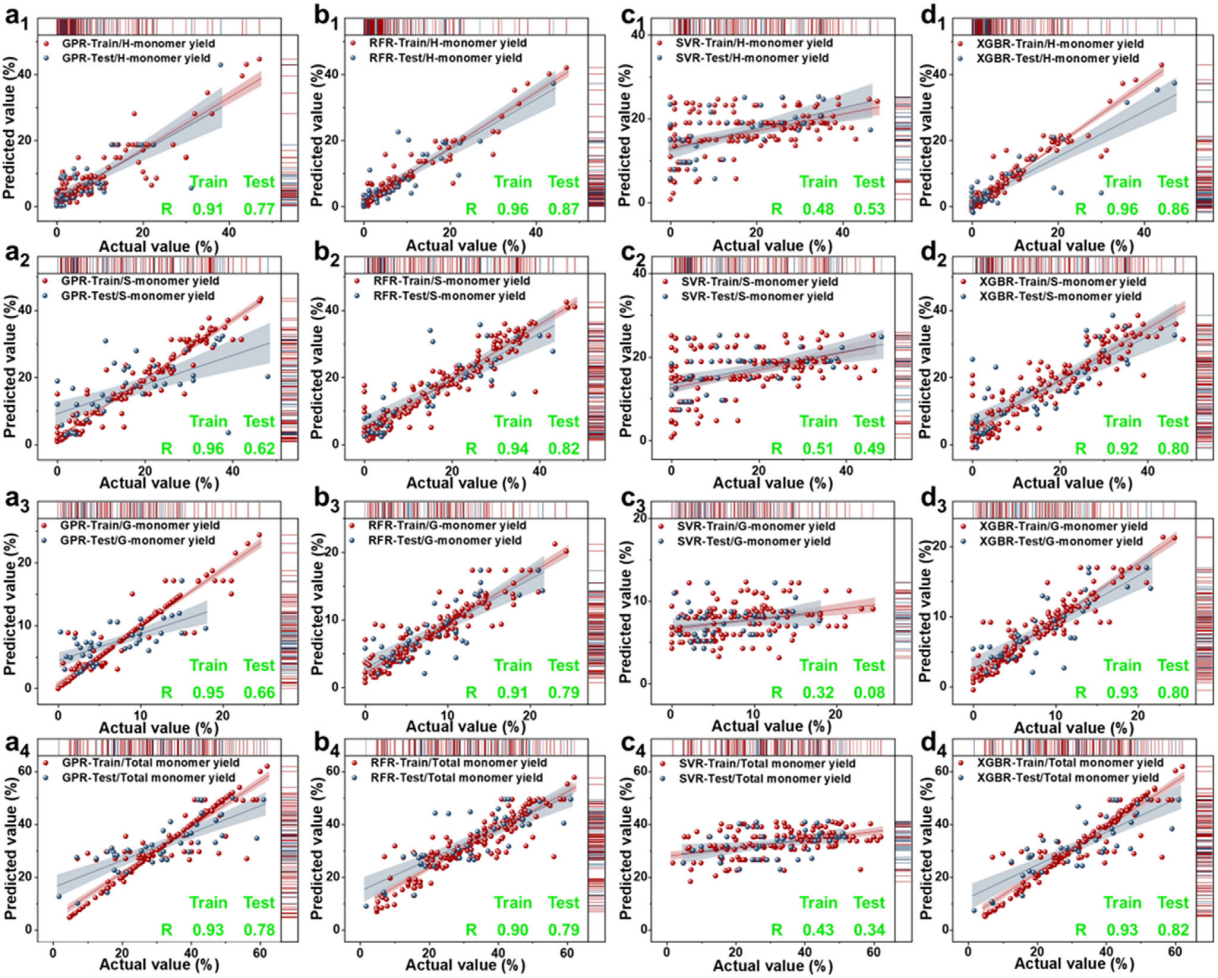
Actual versus predicted data in the 5‐fold cross‐validation for all developed models, including GPR a), RFR b), SVR c), and XGBR d) for H‐, S‐, G‐, and total monomer (1–4). The training and testing datasets are represented by distinct markers, with corresponding R values provided for both sets.

The ML models were evaluated based on their ability to predict the yields of monomers in the RCF process, utilizing metrics such as correlation coefficient (R) values, root mean square error (RMSE), and mean absolute error (MAE) (**Figure** **4**; Table S1, Supporting Information). The Extreme Gradient Boosting Regression (XGBR) model demonstrated exceptional accuracy. During the training phase, it achieved R values ranging from 0.92 to 0.96, with RMSE and MAE values between 1.18 and 5.28, and 1.18 to 3.78, respectively. In the testing phase, R values were between 0.80 and 0.86, with RMSE values spanning 3.99 to 8.31, and MAE values from 2.85 to 6.90. These metrics underscore the proficiency of the XGBR model in capturing complex interdependencies among monomer yields. The Gaussian Process Regression (GPR) model also exhibited strong performance, particularly for H‐monomer and total monomer yields. During the training phase, R values ranged from 0.91 to 0.96, indicating a robust correlation between predicted and actual values. However, in the testing phase, the GPR model displayed slightly larger deviations, with R values from 0.62 to 0.78, and higher RMSE and MAE compared to the XGBR model, suggesting limitations in its ability to generalize to unseen data. The Random Forest Regression (RFR) model demonstrated moderate predictive accuracy, benefiting from its ensemble approach but occasionally overfitting the training data. The Support Vector Regression (SVR) model, on the other hand, provided reasonable predictions, striking a balance between bias and variance, though it struggled with scalability on larger datasets.

Visual comparisons between model predictions and actual values show that data points from the XGBR model exhibit a trend closely matching the actual value distribution, indicating strong agreement with experimental data (Figure 4). This clustering highlights the capability of the model to minimize both bias and variance. Conversely, GPR graphs showed reasonable clustering during training but exhibited greater deviations during testing, especially for S‐monomer and total monomer yields, indicating higher variance and reduced stability.

Overall, the XGBR model consistently outperformed the GPR model, particularly in the testing phase, due to its effective use of gradient‐boosting techniques that adeptly capture nonlinear relationships.^[^ ^32^ ^]^ The GPR model, although effective during training, exhibits limited generalization capability, likely due to sensitivity to complex nonlinear relationships.^[^ ^33^ ^]^ These findings underscore the importance of selecting appropriate ML techniques for accurately modeling and predicting the dynamics of biomass catalytic pyrolysis. The robust performance of the XGBR model highlights its potential as a reliable tool for predicting monomer yields in this complex process.

ML models often operate as opaque systems, making it challenging to discern how input features influence outputs. Prior to developing a decision‐making framework with ML, it is vital to assess the sensitivity of input‐output relationships. XGBR, recognized as the most effective algorithm, can be analyzed using Shapley additive explanations (SHAP) analysis to enhance interpretability by exploring the connections between inputs and outputs.^[^ ^34^ ^]^ SHAP provides both local and global interpretability: local interpretability assesses the contribution of each input feature to specific predictions, while global interpretability aggregates absolute SHAP values across the dataset to reveal the overall impact of input features.^[^ ^35^ ^]^

**Figure 5:**
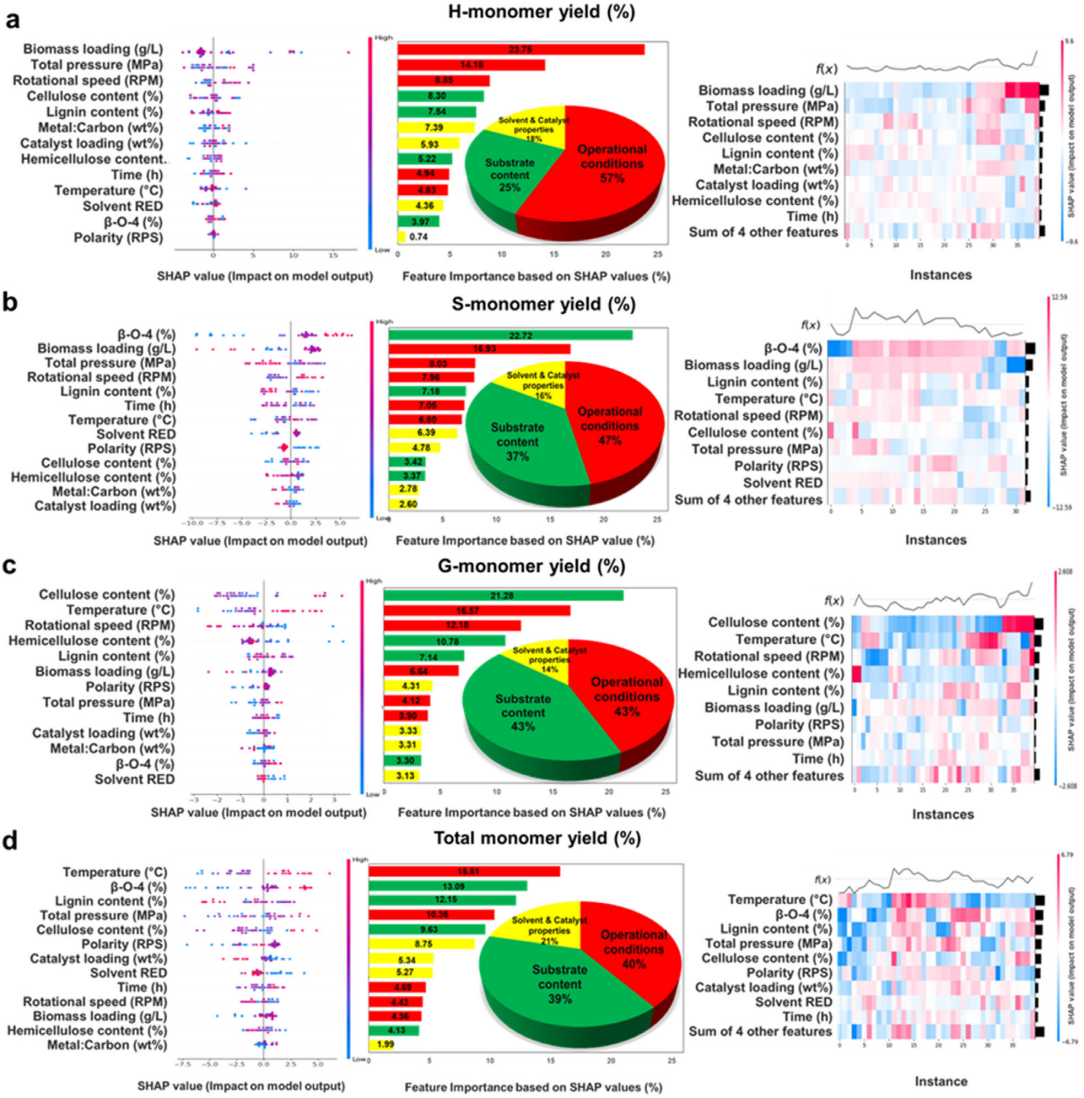
SHAP of developed XGBR for predicting a) H‐, b) S‐, c) G‐, and d) total monomer yields during the RCF process. The left figures depict feature importance based on SHAP values (%), highlighting the relative contribution of each feature to the model predictions. The middle figures illustrate SHAP values (impact on model output) across individual instances, providing insights into how specific features influence predictions for different samples. The right figures summarize the overall feature importance based on SHAP values, offering a comprehensive view of the dominant factors driving the model's performance.

For H‐monomers, biomass loading has the most significant influence, contributing 23.75% to yield improvement, followed by total pressure, which accounts for a 14.18% positive impact. These results emphasize the importance of optimizing biomass input and maintaining appropriate pressure levels to maximize yield (**Figure** **5**a). In the case of S‐monomers, β‐O‐4 linkages are the most critical factor, with a 22.72% contribution, while biomass loading plays a secondary role, contributing 16.93%. This highlights the need to focus on both lignin structure and biomass concentration (Figure 5b). For G‐monomers, cellulose content is the dominant factor, contributing 21.28%, followed by temperature, which accounts for 16.57%, underscoring the necessity of maintaining optimal thermal conditions (Figure 5c). When considering total monomer yield, temperature emerges as the most influential factor (15.81%), followed by β‐O‐4 linkages (13.09%), lignin content (12.15%), and total pressure (10.36%). Across all monomer types, operational conditions, such as temperature and pressure, are the most significant category of influence, accounting for 40–57% of the variability. Substrate content contributes 25–43%, highlighting the importance of selecting biomass with favorable properties and concentrations. Although catalyst and solvent properties are less impactful (14–21%), their careful optimization can still enhance yields significantly (Figure 5d).

The complexity of the RCF process is vividly illustrated through SHAP analysis, which adeptly captures the intricate interplay of factors influencing monomer yields. In contrast to PCA, which struggled to fully encapsulate the multifactorial interactions inherent in the dataset, SHAP analysis excels by quantifying the individual contributions of variables and unraveling their complex interdependencies. These insights emphasize the necessity of a comprehensive optimization strategy that harmonizes operational conditions, substrate content, and catalyst‐solvent interactions. By harnessing the granular understanding provided by SHAP, the yields of H‐, S‐, G‐, and total monomers can be strategically maximized, establishing a robust framework for advancing the efficiency and outcomes of the RCF process.

### 2.3 Optimization and Experimental Verification Results

The best‐developed XGBR model was employed to conduct a multi‐objective optimization process to determine the optimal conditions for maximizing monomer yields. Using the MOPSO algorithm, optimal ranges for substrate content, solvent‐catalyst properties, and operational conditions were established, as detailed in the Supporting Excel File. These conditions were predicted to yield H‐monomer (5.1–9.9%), S‐monomer (23.2–34.9%), G‐monomer (14.3–15.5%), and total monomer (43.2–59%). To validate the model, experiments were conducted for three randomly selected optimal conditions (Figure 5a; Table S2, Supporting Information). The prediction errors for H‐monomers varied across conditions: 22.1% for condition (A), but substantially higher for (B) and (C), at 77.8% and 64.2%, respectively. For S‐monomers, the model performed more consistently, with errors of 14.9% (A), 8% (B), and 17.4% (C). G‐monomer prediction showed high error in condition (A) at 50%, moderate in (B) at 22.4%, and low in (C) at 3.3%. In contrast, total monomer yield predictions were highly accurate across all conditions, with errors of only 2.1% (A), 2.6% (B), and 2.0% (C). These results demonstrate robust predictive accuracy of the ML model for total monomer yields, even in the presence of variability among individual monomer predictions. The low errors in total yield underscore the reliability of the model in optimizing RCF processes, while the observed variability at the individual monomer level indicates potential for further model refinement. This ML framework demonstrates significant potential for advancing sustainable lignin valorization by providing accurate and generalizable predictions within the optimized process space for biorefinery applications.

**Figure 6:**
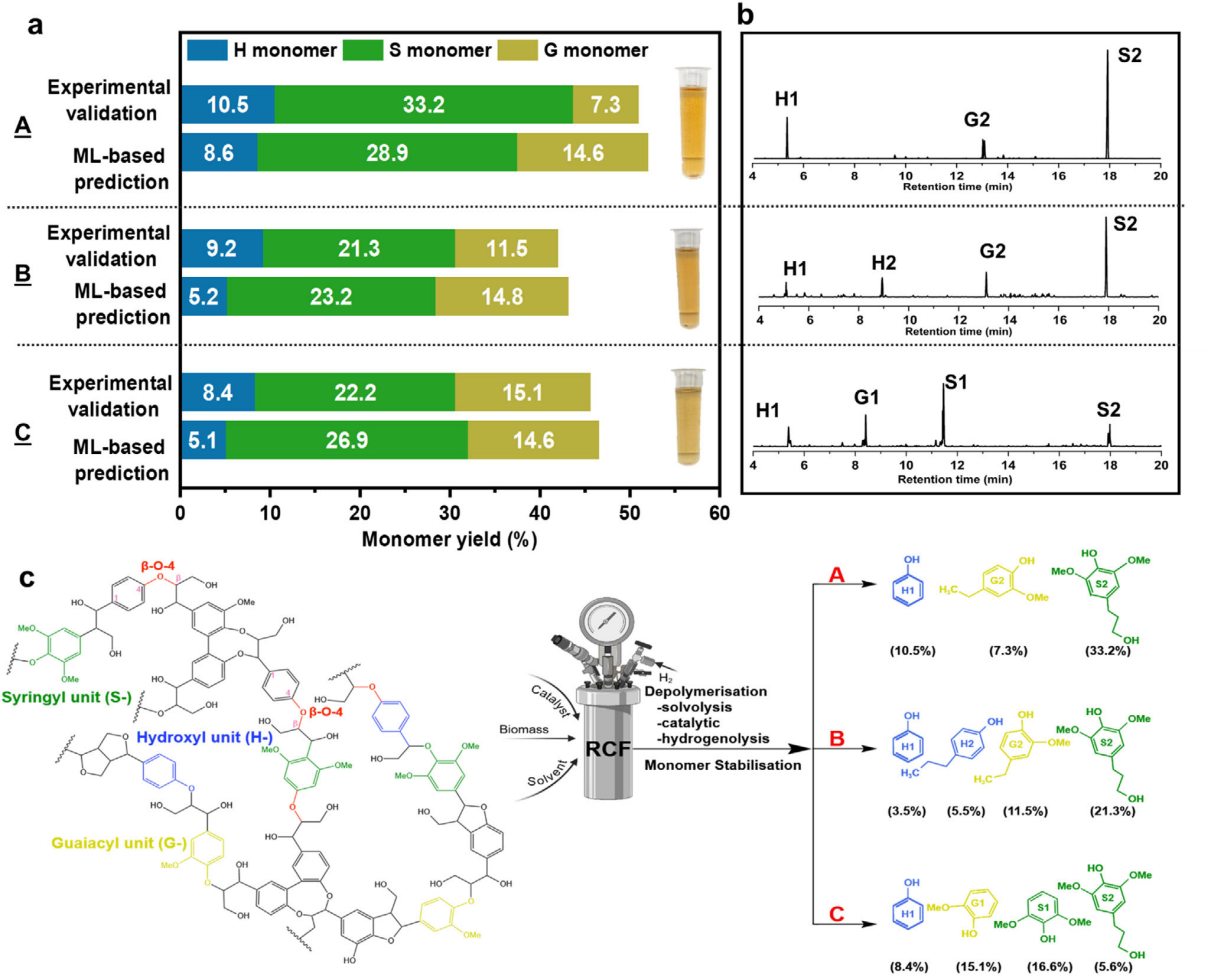
XGBR‐predicted results versus experimental validation results. a) Comparison of H‐, S‐, and G‐monomer yields (%) between ML‐based predictions and experimental validation across three conditions A–C). b) GC spectra of lignin‐derived monomers from three experimental validations. c) Reaction pathways for lignin conversion to monomers under different experimental conditions (A–C). Condition A: Substrate (aspen); Solvent (100 mL: 50 mL water + 50 mL ethylene glycol (EtG)); Catalyst (Ru/C; 5 wt.% metal‐to‐carbon; catalyst loading 2.1 wt.%). Condition B: Substrate (pine); Solvent (100 mL: 100 mL EtG); Catalyst (Ru/C; 10 wt.% metal‐to‐carbon; catalyst loading 2.3 wt.%). Condition C: Substrate (aspen); Solvent (100 mL: 50 mL water + 50 mL butanol); Catalyst (Ru/C; 5 wt.% metal‐to‐carbon; catalyst loading 3.5 wt.%). H1, phenol; H2, propylphenol; S1, syringol; S2, 4‐n‐propanolsyringol; G1, guaiacol; G2, 4‐n‐propanolguaiacol.

The bio‐oil produced under the three experimental validation conditions exhibited selective composition. For condition (A), the bio‐oil contained phenol (H1), 4‐n‐propanolguaiacol (G2), and 4‐n‐propanolsyringol (S2). In condition (B), the primary components were phenol (H1), propylphenol (H2), 4‐n‐propanolguaiacol (G2), and 4‐n‐propanolsyringol (S2). Condition (C) yielded bio‐oil comprising phenol (H1), guaiacol (G1), syringol (S1), 4‐n‐propylsyringol (S2) (**Figure** **6**b). These aromatic and phenolic compounds are valuable intermediates in the synthesis of high‐value chemicals and fuels, such as phenolic resins, vanillin, antioxidants, pharmaceuticals, and drop‐in biofuels. Through catalytic upgrading processes like hydrodeoxygenation or catalytic cracking, these compounds can be transformed into hydrocarbons suitable for gasoline, diesel, or jet fuel, contributing to sustainable energy solutions.^[^ ^36^, ^37^ ^]^ The pathways of lignin conversion into lignin monomers are illustrated in Figure 6c, showcasing the intricate chemical transformations involved. The process begins with solvolysis, depolymerization, and hydrogenolysis, facilitated by solvents such as ethylene glycol (EtG) and butanol, as well as catalysts like Ru/C under hydrogen pressure. EtG enhances hydrolysis yield and monomer production efficiency, while butanol stabilizes the resulting monomers.^[^ ^13^ ^]^ Water also plays a critical role in stabilizing monomers and promoting hydrolysis.^[^ ^38^ ^]^ However, the cleavage of strong β‐O‐4 bonds requires specific conditions, including moderate temperatures (160–170 °C), a catalyst (Ru/C), and hydrogen, which are essential for bond dissociation. This interplay between solvent effects and catalytic conditions underscores the complexity of lignin depolymerization, emphasizing that it is a meticulously controlled process rather than a simple reaction.^[^ ^8^ ^]^

### 2.4 Environmental Impact and Socioeconomic Benefits of the Aspen Biorefinery in China

A multi‐product biorefinery in China, operating at optimal capacity to process 140 million tons of aspen biomass annually, has the potential to produce 12.4 million tons of lignin monomers, 32.7 million tons of xylose, and 60.4 million tons of pulp (**Table** **1**). The significant greenhouse gas (GHG) footprint associated with energy use is primarily due to the reliance on carbon‐intensive resources. Despite China's ongoing efforts to reduce its dependence on fossil fuels, coal and natural gas remain the dominant sources of heat and electricity production.^[^ ^39^ ^]^ To evaluate the environmental benefits of the biorefinery, the well‐to‐wheel GHG emissions of its products were compared with those of the conventional commercial counterparts. All biorefinery products exhibit significantly lower GHG emissions. For example, producing 1 kg of lignin monomers in the biorefinery emits 2.7 kg CO_2_eq, which is 11.5% lower than the 3.05 kg CO_2_eq emitted by fossil‐based products. Similarly, biorefinery‐derived xylose and pulp emit 15.4% and 6.0% less CO_2_eq, respectively, compared to their fossil‐based equivalents (Table 1).

**Table 1:** Annual production of multiple products from 140 million tons of available aspen biomass and the associated well‐to‐wheel GHG emissions.

Products	Annual production, F_i_ [ton]	Weight fraction, X_i_ [kg kg^−1^]	C _well to wheel_ [kg CO_2_ eq kg^−1^ product]	C _well to wheel_ [kg CO_2_ eq kg^−1^ fossil‐based product]
Lignin monomers	12.4 × 10^6^	0.12	2.7	3.05^a)^
Xylose	32.7 × 10^6^	0.31	2.2	2.60^a)^
Pulp	60.4 × 10^6^	0.57	0.74	0.79^a)^
Total	105.5 × 10^6^	1.00		–

^a)^ According to the Ecoinvent 3 database.

**Figure 7:**
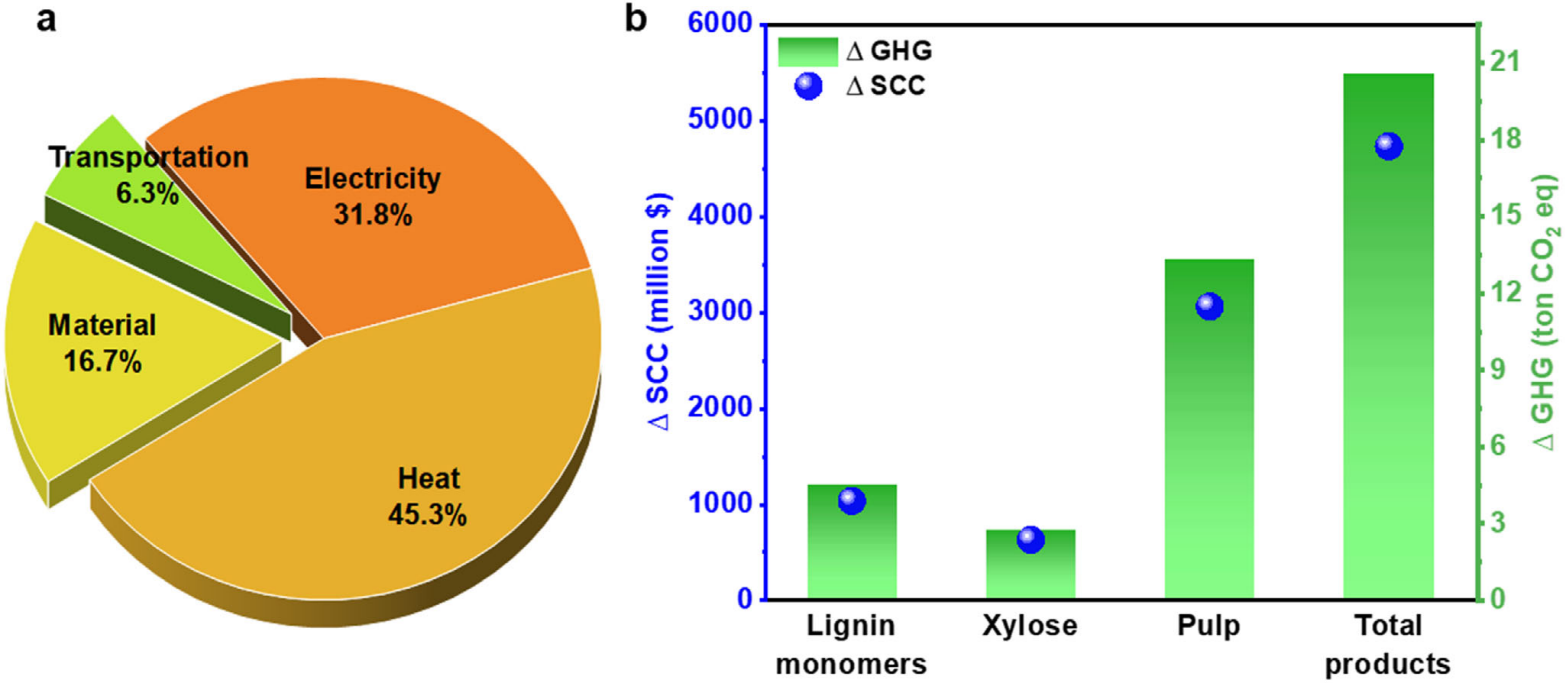
Resource distribution and environmental‐economic impacts of the aspen biorefinery process. a) Contribution of each utilized resource in C_well to wheel_ of the aspen biorefinery. b) ∆_GHG_ and ∆_SCC_ resulting from byproducts produced in the biorefinery process.

**Figure** **7**a illustrates the contribution of various resources to the biorefinery's total GHG emissions. Energy consumption is the largest contributor, accounting for 77.1% of emissions, with electricity and heat generation responsible for 45.3% and 31.8%, respectively. Material inputs contribute 16.7% of total emissions, while transportation plays a relatively minor role at 6.3%. The integration of the biorefinery's products into the industry results in a reduction of GHG emissions (∆_GHG_) and associated social costs (∆_SCC_), highlighting both environmental and economic benefits (Figure 7b). Substituting lignin monomers could prevent 4.5 million tons of GHG emissions annually, resulting in socioeconomic cost savings of ≈$1037 million in socioeconomic costs associated with environmental damage. Replacing traditional commercial products with xylose and pulp from the biorefinery reduces 2.7 and 13.3 million tons of CO_2_eq, respectively. Collectively, the contributions of all biorefinery products can achieve a total reduction of 20.6 million tons of CO_2_eq in GHG emissions and $4729 million in related social costs. Previous studies have emphasized the potential of biorefineries to mitigate GHG emissions, with factors such as feedstock availability, biorefinery location, and the diversity of products influencing their impact. For example, converting corn stover in Iran into multiple products, such as ethanol and biodiesel, reduced emissions by 4.3 million tons of CO_2_eq.^[^ ^40^ ^]^ Similarly, refining one ton of apple pomace into mycoprotein and biogas was projected to decrease emissions by 2.5 tons of CO_2_eq.^[^ ^41^ ^]^

In summary, the comprehensive utilization of aspen biomass within a multi‐product biorefinery framework demonstrates significant potential for reducing GHG emissions compared to traditional commercial products. By maximizing the production of high‐value goods, the biorefinery contributes to environmental sustainability while delivering considerable socioeconomic benefits. This underscores the importance of multi‐product biorefineries as a key strategy for advancing a low‐carbon economy, promoting sustainable development, and reducing reliance on non‐renewable resources.

The biorefinery with an annual processing capacity of 660 kt per year necessitates an initial capital expenditure (CAPEX) of $315.2 million. This substantial investment of $315.2 million is allocated to cover various critical components, e.g., RCF area for reactor expenses ($122.7 million), RCF area for non‐reactor costs ($40.1 million), storage facilities, and essential utilities (Table S3, Supporting Information). The annual operational expenditures (OPEX) is $293.3 million, with the primary cost drivers including material expenses (*for instamce*, aspen, ethylene glycol, and hydrogen), utility requirements (electricity and heat), and additional operational expenditures such as labor costs ($35.2 million), maintenance ($8.5 million), and fixed charges ($29.3 million). These recurring expenditures represent the financial obligations associated with the plant's daily operations and its commitment to maintaining functional equipment and retaining skilled personnel. After accounting for all costs, the plant yields an annual net cash flow (Total Revenues – OPEX) of $103.5 million. This positive financial outcome suggests that the plant is capable of producing a significant return on investment, highlighting its overall profitability and operational success. With an NPV of $332.9 million, the project indicates its potential to generate returns exceeding the initial $315.2 million capital investment when evaluated at a 15% discount rate. This positive Net Present Value (NPV) indicates that the plant's future cash flows are sufficiently robust to justify the upfront investment, signaling a promising and profitable project. The project's 32.8% Internal Rate of Return (IRR) surpasses the 15% discount rate, indicating its financial attractiveness and viability as a profitable investment opportunity.

## 3 Conclusion

This study presents an ML‐driven framework for optimizing the RCF of LCB, enabling efficient production of high‐value lignin monomers. By integrating a comprehensive dataset compiled from numerous experimental studies, the developed models, particularly XGBR, demonstrated strong predictive performance and generalizability. Feature importance analysis identified operational parameters as the dominant factors influencing monomer yields, followed by substrate composition and catalyst–solvent properties. Experimental validation confirmed the model's robustness, especially in predicting total monomer yields. Scaling the optimized process to an industrial level using Aspen biomass could significantly reduce CO_2_ emissions and generate substantial socioeconomic benefits, underscoring the environmental and economic viability of the approach. Overall, this ML‐enhanced strategy offers a powerful, scalable tool for guiding data‐driven process optimization in lignin valorization. It provides actionable insights for the design of sustainable, low‐carbon biorefineries and represents a significant step toward realizing a carbon‐neutral bioeconomy.

Despite the promising performance shown by the proposed ML framework, several limitations should be acknowledged. The accuracy of the model depends on the quality and diversity of the literature‐derived dataset and may decrease under conditions not well captured in the training data. Selective prediction of individual monomer types (H‐, S‐, G‐) remains challenging, reflecting the inherent complexity of lignin depolymerization pathways. Improving the reliability of such predictions may require more focused, monomer‐specific experimental datasets or the integration of mechanistic knowledge into ML strategies. Although the model was evaluated through cross‐validation and experimental testing under three optimized conditions, additional validation using standardized external datasets or independently generated experimental data would further support its generalization potential. Extending the framework to cover a wider range of biomass types, catalyst systems, or continuous‐flow processing methods could also enhance applicability across real‐world biorefinery applications.

## 4 Experimental Section

### Research Methodology

The process started with an extensive examination of existing literature on monomeric lignin production from LCB through RCF. A database was compiled by selecting studies based on their titles, followed by further filtering to include only those with relevant and complete data. The extracted data were organized into a dataset, which underwent preprocessing steps such as removing outliers and addressing missing values to produce a clean and reliable dataset for ML analysis. Four advanced ML model methods, e.g., GPR, RFR, SVR, and XGBR, were employed to simulate the monomeric lignin yields. The models were fine‐tuned through hyperparameter optimization to improve their accuracy and evaluated using statistical indicators such as the R, RMSE, and MAE (Figure S2a, Supporting Information). The most effective model was then used for feature importance analysis to pinpoint the critical input parameters influencing the results and to optimize the process conditions for better RCF performance. Then, experimental trials were carried out to validate the predictions generated by the robust ML model (Figure S2b, Supporting Information). Finally, a comprehensive socioeconomic and environmental impact analysis was conducted to evaluate the feasibility and sustainability of scaling up the RCF process (Figure S2c, Supporting Information).

### Literature Survey and Variable Identification

To construct the dataset, a systematic search was conducted on the Scopus database using the most relevant keywords, e.g., “Reductive Catalytic Fractionation of Lignocellulosic Biomass,” “Lignin Monomer Production from Biomass,” and “Lignin Depolymerization via Catalytic Hydrogenation.” The search was restricted to research studies published between 1941 and 2024 and focused on titles, abstracts, and keywords to ensure precise and relevant results. This initial query yielded 2321 studies. These results were then manually filtered by reviewing the titles and abstracts to assess their alignment with the research objectives, reducing the selection to 569 studies. A detailed evaluation of these shortlisted studies was subsequently performed to extract the essential data required for dataset construction. Variables were selected carefully based on their significance to the study, ensuring the avoidance of multicollinearity while capturing sufficient variability and quantitative range. The data extraction process involved identifying and organizing key patterns into a structured dataset, as detailed in the Supporting Excel File. To enhance the reliability of the data, statistical outliers were identified and addressed, minimizing potential bias before proceeding to the modeling phase. Lastly, 3451 unique data points were extracted from 54 studies, providing a robust foundation for statistical and ML analyses. Thirteen independent input variables were categorized into four groups: 1) substrate content (cellulose (%), hemicellulose (%), lignin (%), and β‐O‐4 content), 2) solvent properties (polarity (RPS) and solvent RED), 3) catalyst characteristics (metal‐to‐carbon ratio (wt.%) and catalyst loading (wt.%)), and 4) operational conditions (reaction time (h), temperature (°C), total pressure (MPa), biomass loading (g L^−1^), and RPM). The target outputs included the yields of H‐, S‐, G‐, and total monomers (%). These metrics were extracted directly from numerical tables or digitized from graphical data using the GetData software when necessary.

### Modeling Process and Feature Importance Analysis: ML Modeling

To ensure uniformity across all features, input and output variables were normalized, standardizing their scales to prevent any single feature from exerting undue influence on the model's predictions. The dataset was split into two subsets, with 80% allocated for training and 20% reserved for testing. To improve generalization and mitigate the risk of overfitting, a 5‐fold cross‐validation strategy was employed. This process involved dividing the training data into five subsets, where each subset was alternately used as a validation set while the remaining subsets were used for training.^[^ ^42^ ^]^ This iterative approach was applied to several ML algorithms, including GPR, RFR, SVR, and XGBR. After cross‐validation, the models were tested on the reserved test set. Hyperparameter tuning for each algorithm was conducted using the Grid Search method, which systematically explored various combinations of hyperparameters to identify the optimal configuration. The performance of the model was evaluated using key metrics: R, RMSE, and MAE. These metrics offer a comprehensive assessment of predictive capabilities across both training and testing phases of the models.

GPR was a flexible supervised ML method used to model unknown functions based on training data. One of its key strengths was the ability to provide confidence intervals for predictions, offering a clear measure of uncertainty. GPR operates on a nonparametric probabilistic kernel framework, treating input vectors as probability distributions. Instead of using simple scalar values for mean and variance, it computes a mean vector and a covariance matrix to better capture relationships. This approach requires only hyperparameter tuning and does not impose restrictions on the search space during optimization.^[^ ^33^ ^]^ Due to its versatility, GPR was widely applicable to various regression and prediction tasks.^[^ ^43^ ^]^

RFR was an ensemble learning method that enhances prediction accuracy by combining multiple decision trees.^[^ ^44^ ^]^ Each tree operates as an independent regression model, and its outputs are averaged to produce the final prediction. To improve model performance, RFR employs bagging, where multiple datasets are generated through random sampling with replacement, ensuring each tree was trained on a unique subset of the data. A key feature of RFR was its use of randomness in feature selection. At each node, a random subset of predictors was evaluated to determine the best split, which reduces overfitting and increases model robustness. This process continues until the trees are fully grown, with splits recursively partitioning the data at each node. By combining the outputs of all trees and introducing variability through random sampling and feature selection, RFR effectively captures complex patterns in data, making it well‐suited for both linear and nonlinear regression tasks.^[^ ^45^ ^]^

SVR was a supervised ML algorithm designed for predicting continuous numerical data. Its primary objective was to identify a hyperplane in an *n*‐dimensional space that best fits the data while maintaining a margin of tolerance, known as the epsilon‐insensitive zone, around the hyperplane. This margin allows the model to disregard minor deviations and focus on significant discrepancies. The balance between reducing prediction errors and maintaining model simplicity was controlled by a penalty parameter, which ensures an optimal trade‐off between accuracy and generalization. A key strength of SVR lies in the use of kernel functions, which map input data into a higher‐dimensional space where linear separation becomes feasible. Common kernel types include linear, polynomial, radial basis function (RBF), and sigmoid, allowing SVR to effectively model both linear and nonlinear relationships. This adaptability, combined with careful tuning of its parameters, enables SVR to uncover complex patterns in data and deliver precise, reliable predictions across a wide range of regression tasks.^[^ ^46^ ^]^

XGBR was a powerful and efficient ML algorithm based on the gradient boosting framework, designed for regression tasks. It builds an ensemble of decision trees sequentially, where each tree corrects the errors of the previous ones, optimizing the model by minimizing a specified loss function, such as mean squared error. XGBR was highly versatile and capable of capturing both linear and nonlinear relationships in data. It incorporates advanced techniques like regularization (L1 and L2) to prevent overfitting, tree pruning to reduce complexity, and parallel processing for faster computation. It also handles missing values effectively and supports efficient data splitting, even for large datasets.^[^ ^32^ ^]^ With hyperparameters, for instance, learning rate, tree depth, and the number of estimators, XGBR can be fine‐tuned to achieve high accuracy and adaptability, making it a popular choice for diverse regression problems.

### Model Interpretation

Model interpretability was critical as it clarifies how input variables influence the predictions, thereby fostering transparency and trust in decision‐making processes. A prominent method for achieving interpretability was SHAP, a technique grounded in game theory and introduced by Lundberg and Lee.^[^ ^47^ ^]^ SHAP assigns a numerical value to each feature, representing its contribution to the prediction. It conceptualizes features as collaborators, much like players dividing a reward, and calculates their individual impacts on the final output. By highlighting the relative importance of features, SHAP provides a detailed understanding of what drives the model's outcomes.^[^ ^48^ ^]^ This interpretability was particularly advantageous for domain experts, as it helps identify the key factors influencing predictions, enabling more informed and reliable decisions. The data from the robust model were utilized for SHAP analysis to assess the significance of variables, including substrate content, solvent and catalyst properties, and operational conditions, in predicting monomeric lignin yields. Visualizations such as Beeswarm plots, bar charts of mean absolute SHAP values, and pie charts effectively showcased the contribution of each variable, further enhancing the interpretability of the model.

### Optimization Process

Optimizing the hyperparameters of ML models was a powerful approach to enhancing their predictive performance. In this study, a grid search algorithm was chosen to optimize the hyperparameters. The XGBR, identified as the best‐performing model, was selected for further optimization. Its outputs (objective functions) were used as input for the MOPSO algorithm to refine the process. The optimization aimed to identify the optimal input variable conditions that maximize S‐, G‐, and total monomer yields while minimizing H‐monomer yield.

### Validation Process

The predictive accuracy of the ML model was assessed through experimental testing of three randomly selected optimal conditions identified by the algorithm. The experimental parameters, including substrate content, solvent‐catalyst properties, and operational conditions, are documented in Table S2 (Supporting Information). Experimental procedures were executed in a high‐pressure reactor (TK‐PCGF‐2‐250, Mojina Instrument Factory Co., Ltd., Xian, China). Upon reaction completion, phase separation was achieved through filtration to separate the solid and liquid fractions. The liquid phase was subjected to selective extraction using a certain amount of dichloromethane (DCM) to extract the bio‐oil.

Analysis of quantitative bio‐oil composition was performed via gas chromatography‐mass spectrometry (Agilent 7890B GC‐MS) utilizing a non‐polar HP5‐MS (5% phenyl‐methylpolysiloxane) capillary column with ultra‐high purity helium (99.99%) as the mobile phase. The chromatographic method employed the following thermal gradient protocol: initial isothermal period at 50 °C (60 s), followed by a linear temperature progression at 15 °C min^−1^ to 300 °C, with a final isothermal hold period of 420 s. The injection port and MS transfer line were maintained at 250 and 290 °C, respectively. The compositional analysis of raw and pretreated pulp was conducted through standardized NREL protocols employing sequential acid hydrolysis methodology.^[^ ^49^ ^]^

### Socioeconomic, Environmental, and Economic Impact Analysis_Socioeconomic Analysis

This analysis was conducted using experimental data obtained from laboratory‐scale experiments, as illustrated in the mass balance provided in Figure S3 (Supporting Information). Equations ((1), (2), (3), (4), (5)) were employed to quantify the positive impacts of refining aspen biomass into multi‐bioproducts (lignin monomers, xylose, and pulp), focusing on the reduction of GHG emissions and associated social costs. To estimate the maximum annual availability of aspen biomass (F_AB_) for collection in China, a value of 140 million tons was adopted.^[^ ^50^ ^]^ The annual mass of each product was determined using Equation (1), which incorporates the optimal yields achieved during the refining process.

(1) $$F_{\mathrm{bi}}=Y_{i}\times F_{\mathrm{AB}}$$

Here, F_bi_ (ton) represents the annual mass of each product generated, while Y_i_ (ton/ton) denotes the yield of each product in its respective process. For this analysis, it was assumed that each product would serve distinct markets. For instance, the lignin monomers could serve as sustainable alternatives to fossil‐derived phenol, while the xylose and pulp could replace commercially available xylose and pulp, respectively.

The reduction in GHG emissions associated with each product substitution (∆_GHGi_, ton CO_2_eq) was calculated using Equation (2). This reduction was derived by comparing the baseline emissions (F_bi_ × C_i_) associated with the annual consumption of conventional or fossil‐based products with the emissions resulting from the corresponding biorefinery products.^[^ ^51^ ^]^

(2) $$\Delta_{\mathrm{GH}G_{i}}=F_{b_{i}}\times \left(C_{i\;}-C_{b_{i}}\right)$$

where, F_bi_ (ton) represents the mass of the bio‐based product generated in the biorefinery, while C_bi_ and C_i_ (kg CO_2_eq kg^−1^ bioproduct), denote the well‐to‐wheel GHG emission factors for biorefinery products and conventional products, respectively. The total GHG emissions reduction achieved by introducing all biorefinery products to the market was then calculated using Equation (3);^[^ ^52^ ^]^

(3) $$\Delta_{\mathrm{GHG}}=\sum \Delta_{\mathrm{GH}G_{i}}$$

Finally, the reduction in social cost (∆_SCC_, $) associated with the decrease in carbon emissions was computed using Equations (4 and 5);^[^ ^53^ ^]^

(4) $$\Delta_{\mathrm{SCC}}=\sum \Delta_{\mathrm{SC}C_{i}}$$

(5) $$\Delta_{\mathrm{SC}C_{i}}=\Delta_{\mathrm{GH}G_{i}}\times \mathrm{SCC}$$

Here, ∆_SCC__i_ is the reduction in social cost attributed to each product's contribution to the market, while *SCC* ($ t^−1^ CO_2_) is the social cost of carbon, which estimates the economic damages associated with emitting one additional ton of GHG (CO_2_ equivalent) into the atmosphere. These damages encompass various factors, such as environmental degradation, health impacts, and economic disruptions caused by climate change. The SCC was typically calculated by governments and can be influenced by several parameters, including time horizon, socioeconomic factors, climate sensitivity, discount rate, and emission trajectories. According to the Environmental Protection Agency of the United States (EPA),^[^ ^54^ ^]^ the SCC was estimated to range between 120 and 340 (US$ t^−1^ CO_2_). In this study, the reduction in SCC was calculated using the average economic damages, which was ≈230 US$ t^−1^ CO_2_.

### Socioeconomic, Environmental, and Economic Impact Analysis_Well‐to‐Wheel GHG Emissions

To ensure a comprehensive assessment of the environmental impact of the entire system, well‐to‐wheel GHG emissions were calculated. This approach accounts for the contributions of various GHGs, providing a more accurate understanding of the system's overall environmental footprint. A reference unit of 1 ton of aspen biomass was used as the basis for the analysis, with all energy consumption, material inputs, waste generation, and transportation metrics based on this unit. The system boundary for the well‐to‐wheel analysis is illustrated in Figure S4 (Supporting Information). Scaling methods proposed by Piccinno et al.^[^ ^55^ ^]^ were employed to extrapolate laboratory data for estimating the energy consumption of equipment. Table S2 (Supporting Information) provides an overview of the quantities of consumed resources and their corresponding GHG emissions, with data sourced from the Ecoinvent 3 database. Well‐to‐wheel GHG emissions (C_total_, kg CO_2_ eq) were calculated using Equation (6), which involves multiplying the quantity of each consumed resource (*Q_consumed resource_*) by its corresponding GHG emissions (*C_consumed resource_*) and then summing these values.

(6) $$C_{\mathrm{total}}=\sum Q_{\mathrm{consumed}\;\mathrm{source}}\times C_{\mathrm{consumed}\;\mathrm{resource}}$$

### Socioeconomic, Environmental, and Economic Impact Analysis_Process Economics

For companies, the decision to invest in new industrial processes or facilities depends on the economic viability of such projects. The financial feasibility of this biorefinery was evaluated using NPV and IRR. The NPV, defined by Equation (7), calculates the present value of future cash flows derived from the initial CAPEX. A positive NPV indicates a profitable investment, while a negative NPV suggests that the project was not financially viable. The IRR, defined by Equation (8), represents the discount rate at which the NPV equals zero. An IRR exceeding the discount rate indicates a profitable project.^[^ ^56^ ^]^

(7) $$\mathrm{NPV}=\sum_{t=1}^{n}\frac{CF_{t}}{{\left(1+i\right)}^{t}}\;-\mathrm{CAPEX}$$

(8) $$0=\sum_{t=1}^{n}\frac{CF_{t}}{{\left(1+\mathrm{IRR}\right)}^{t}}\;-C$$

Here, CF_t_ represents the net cash flow in year *t*, calculated as the difference between discounted annual revenues and OPEX. A discount rate (*i*) of 15% was used to estimate the present value of future cash flows. The analysis assumes an initial CAPEX in year 0 and a project lifespan of 20 years (*n*). A key challenge in estimating CAPEX for projects at low Technology Readiness Levels (TRLs) was the lack of reliable cost data for specific production capacities or equipment dimensions. For the RCF biorefinery, the exact scaling factor remains undetermined. To address this uncertainty, data from cited references were used to estimate the total equipment costs for a biorefinery with an annual production capacity of 660,000 tons.^[^ ^57^ ^]^ The cost distribution among sub‐modules (for instance, the RCF area and utilities) is detailed in Table S4 (Supporting Information). The annual OPEX, which accounts for the costs of manufacturing and selling the products, is outlined in Table S4 (Supporting Information). The selling price of pulp was based on the market price of bleached sulfate pulp in China. For lignin monomers, a price of 1750 $ ton^−1^ was assumed, which reflected an estimated average market price within the 1500–2000 $ ton^−1^ range, comparable to the market value of phenol‐formaldehyde resins.^[^ ^56^ ^]^ Given the high impurity levels in the xylose produced by this biorefinery, the lowest market price for xylose was used as the basis for economic calculations.

## Conflict of Interest

The authors declare no conflict of interest.

## Supporting information



Supporting Information

Supplementary File 1

## Data Availability

The data that support the findings of this study are available in the supplementary material of this article.
