# Correction to “Scalable Lignin Monomer Production Via Machine Learning‐Guided Reductive Catalytic Fractionation of Lignocellulose”

**DOI:** 10.1002/advs.75953

**Published:** 2026-06-02

M. Madadi, E. Kargaran, S. S. Hashemi, C. Sun, J. F. Denayer, K. Karimi, F. Sun, V. K. Gupta, “Scalable Lignin Monomer Production Via Machine Learning‐Guided Reductive Catalytic Fractionation of Lignocellulose,” *Advanced Science* 12, no. 42 (2025): e10496, https://doi.org/10.1002/advs.202510496.

In the published article, the individual contributions of xylose and pulp to greenhouse gas emission reduction and social cost savings were inadvertently interchanged in Figure 7B and in the corresponding sentence in the Environmental Impact section.

This error was due to a labeling error in the figure and narrative. It does not affect the total reported GHG reduction, the total social cost savings, the calculation methodology, or the overall conclusions of the article.

The sentence in the Environmental Impact section should be corrected from (changes in bold):

“Replacing traditional commercial products with xylose and pulp from the biorefinery reduces **2.7** and **13.3 million tons of CO_2_‐eq**, respectively”.

to:

“Replacing traditional commercial products with xylose and pulp from the biorefinery reduces **13.3** and **2.7 million tons of CO_2_‐eq**, respectively”.

The original and corrected versions of Figure 7B are provided below.


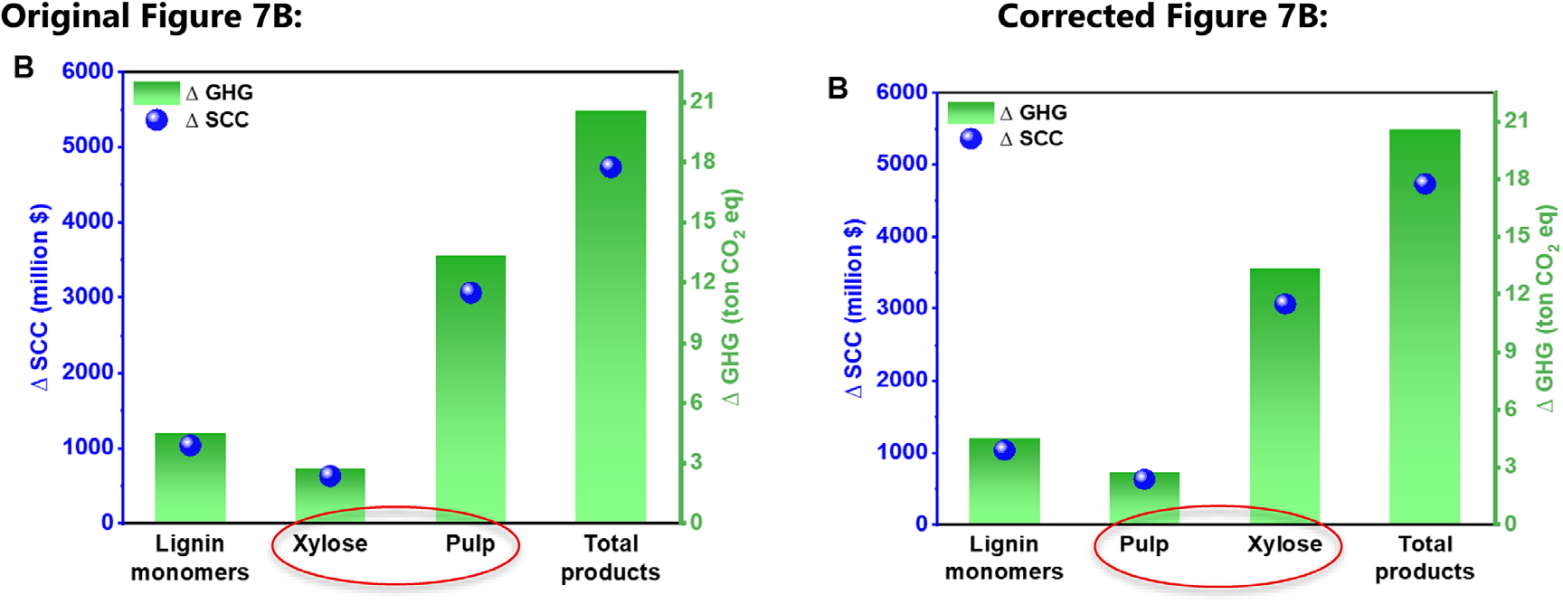


The authors apologize for this error and any confusion it may have caused.

