# Supplementary material for: Scalable Lignin Monomer Production Via Machine Learning‐Guided Reductive Catalytic Fractionation of Lignocellulose
Source: Adv Sci (Weinh). 2025 Aug 27;12(42):e10496. doi: 10.1002/advs.202510496 (PMC12622498; doi:10.1002/advs.202510496)
Supplement: Supplementary file 1 — Supporting Information [file ADVS-12-e10496-s001.docx]

***Supporting Information***

**Scalable Lignin Monomer Production *via* Machine Learning-Guided Reductive Catalytic Fractionation of Lignocellulose**

Meysam Madadi^a, 1^, Ehsan Kargaran^a,1^, Seyed Sajad Hashemi^a^, Chihe Sun^a^, Joeri F.M. Denayer^b^ , Keikhosro Karimi^b,*^, Fubao Sun^a,*^, Vijai Kumar Gupta^c,*^

*^a^ Key Laboratory of Industrial Biotechnology, Ministry of Education, School of Biotechnology, Jiangnan University, Wuxi 214122, China*

*^b^ Department of Chemical Engineering, Vrije Universiteit Brussel, 1050, Brussels, Belgium*

*^c^ School of Biotechnology, Dublin City University, Glasnevin, Dublin, D09 K20V, Ireland*

* Corresponding Author

Keikhosro Karimi ([Keikhosro.Karimi@vub.be](mailto:Keikhosro.Karimi@vub.be))

Fubao Sun ([fubaosun@jiangnan.edu.cn](mailto:fubaosun@jiangnan.edu.cn))

Vijai Kumar Gupta ([vijaikumar.gupta@dcu.ie](mailto:vijaikumar.gupta@dcu.ie))

^1^ The two authors have the same contribution to this study

**Supplementary figures**


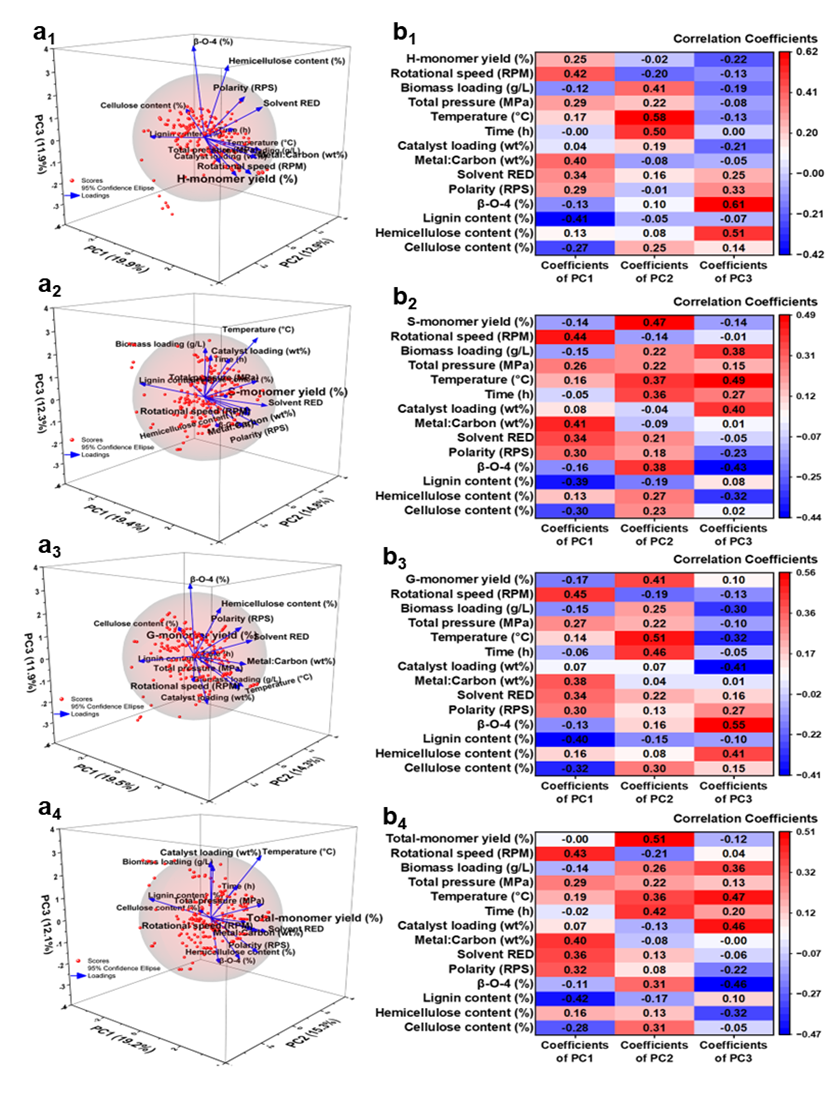


**Fig. S1** PCA of the compiled dataset: Effects of input variables on H- (a_1_), S- (a_2_), G- (a_3_), and total monomers yields (a_4_) and correlation of input variables with the top-three components (b_1_-b_4_)


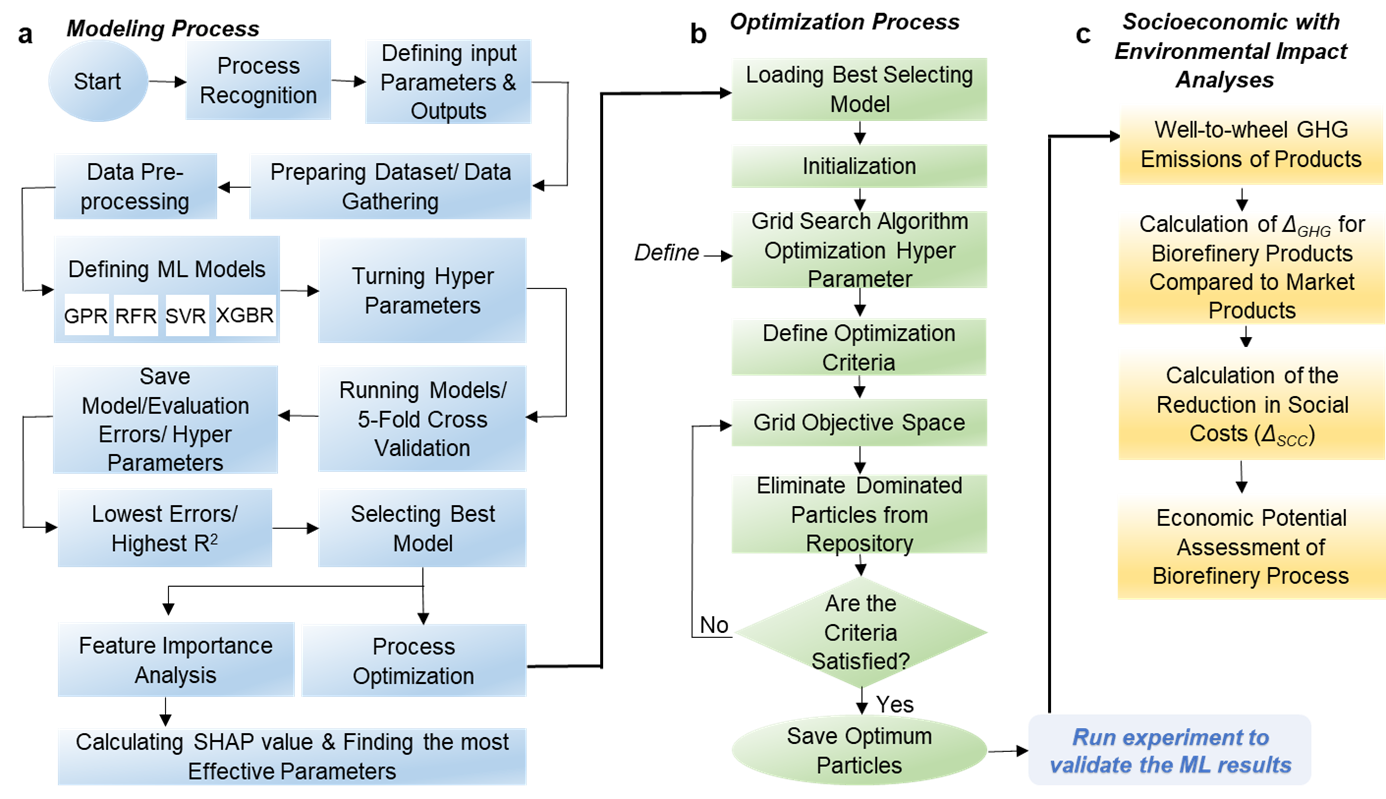


**Fig. S2.** Integrated process workflow of ML modeling, optimization, and impact analysis: A structured diagram outlining the (a) ML modeling, (b) optimization process, and (c) socioeconomic and environmental impact analyses applied in this study.


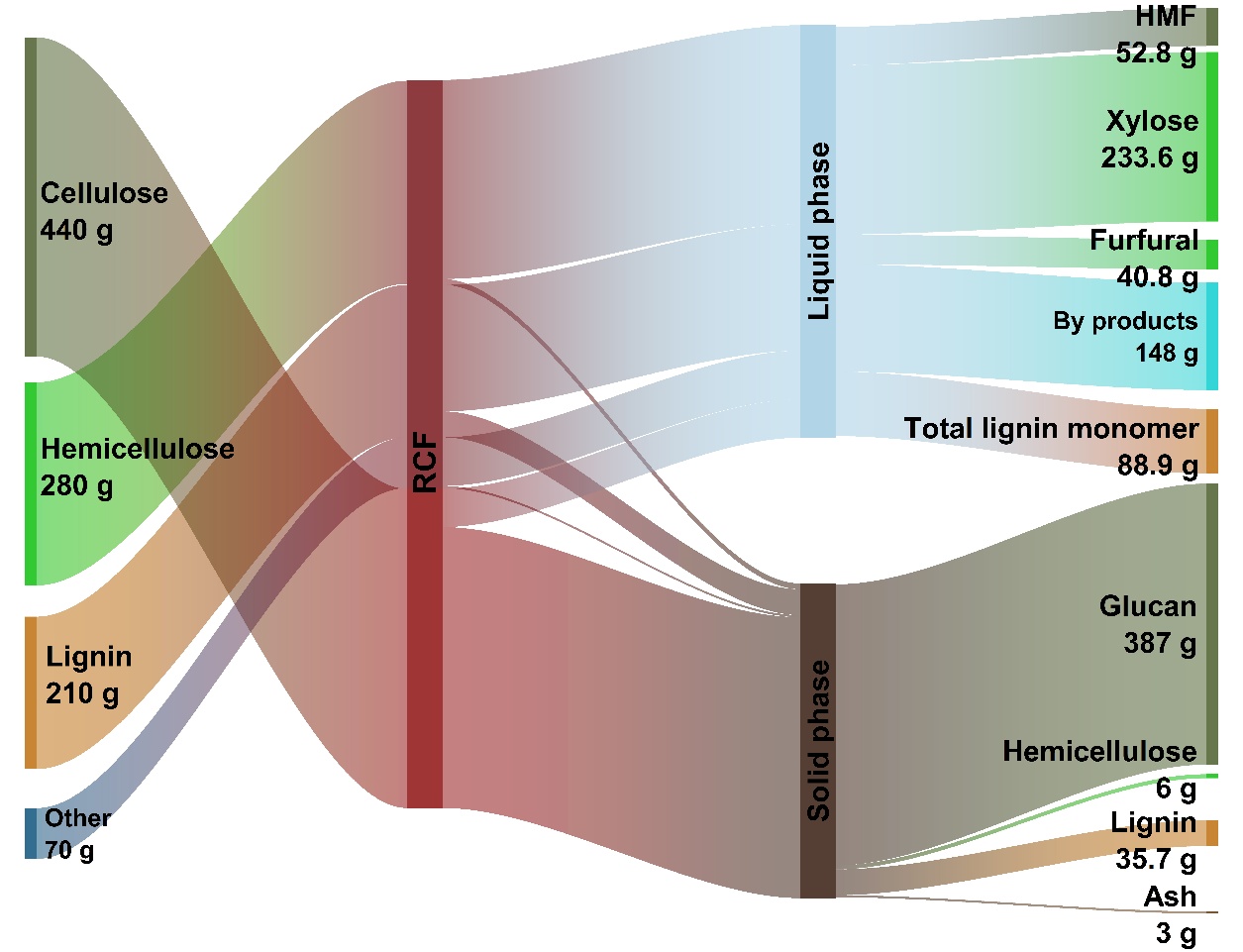


**Fig. S3** The mass balance of the aspen biomass biorefinery process based on 1000 g of aspen biomass valorization to produce *lignin oil, xylose*, and *solid pulp*. The *RCF conditions* were based on ***condition (A) in Table S1***

**
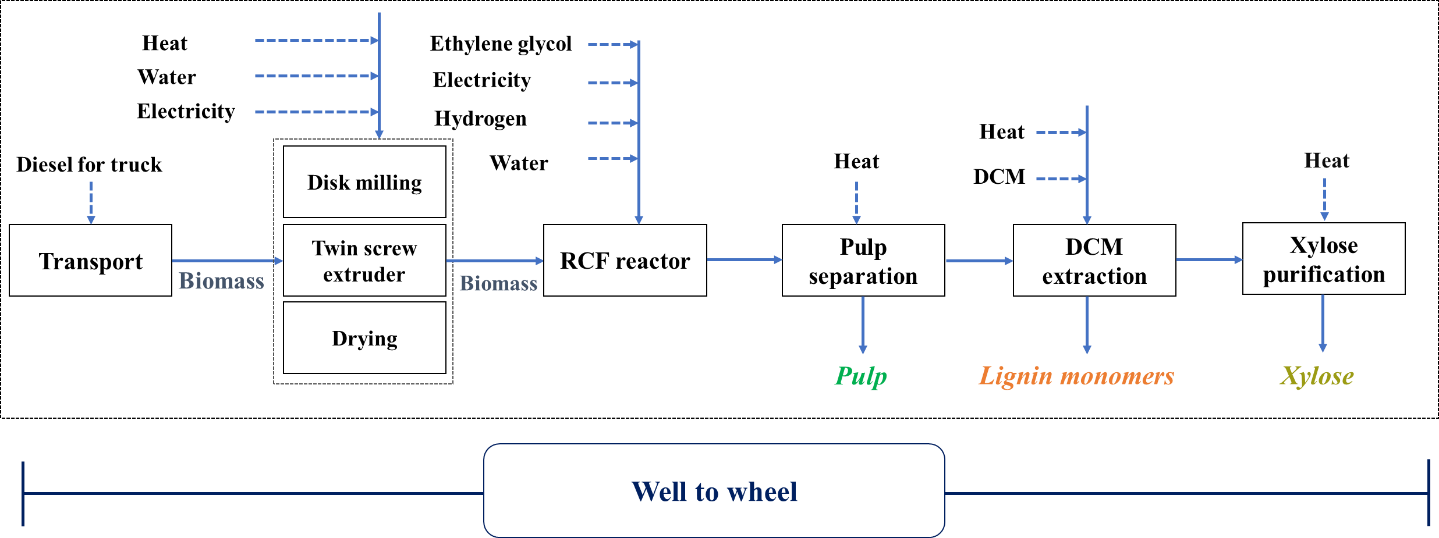
**

**Fig. S4** RCF’s system boundary for well-to-wheel analysis of aspen wood processing into pulp, lignin monomers, and xylose-rich products.

**Supplementary Tables**

**Table S1** Statistical factors of the ML models for predicting H-, S-, G-, and total monomer yields

| Model Types | | | | | | | | | | |
| --- | --- | --- | --- | --- | --- | --- | --- | --- | --- | --- |
| Output  targets | Statistical factors | GPR | | RFR | | SVR | | XGBR | |  |
|  |  | Train | Test | Train | Test | Train | Test | Train | Test |  |
| H-monomer yield (%) | R | 0.91 | 0.77 | 0.96 | 0.87 | 0.48 | 0.53 | 0.96 | 0.86 |  |
|  | RMSE | 3.86 | 5.47 | 2.64 | 4.58 | 11.48 | 10.53 | 2.31 | 6.10 |  |
|  | MAE | 2.35 | 3.45 | 7.33 | 3.10 | 9.73 | 8.68 | 1.18 | 3.38 |  |
| S-monomer yield (%) | R | 0.96 | 0.62 | 0.94 | 0.82 | 0.51 | 0.49 | 0.92 | 0.80 |  |
|  | RMSE | 3.43 | 10.06 | 4.38 | 7.25 | 11.38 | 11.10 | 5.28 | 7.68 |  |
|  | MAE | 2.23 | 6.76 | 2.94 | 5.41 | 9.53 | 9.38 | 3.78 | 5.77 |  |
| G-monomer yield (%) | R | 0.95 | 0.66 | 0.91 | 0.79 | 0.32 | 0.08 | 0.93 | 0.80 |  |
|  | RMSE | 1.64 | 3.50 | 2.20 | 3.23 | 5.24 | 4.66 | 1.75 | 3.99 |  |
|  | MAE | 0.64 | 2.83 | 1.51 | 2.52 | 4.05 | 3.78 | 1.25 | 2.85 |  |
| Total monomer yield (%) | R | 0.93 | 0.78 | 0.90 | 0.79 | 0.43 | 0.34 | 0.93 | 0.82 |  |
|  | RMSE | 5.11 | 9.08 | 6.08 | 8.80 | 12.92 | 4.66 | 5.14 | 8.31 |  |
|  | MAE | 2.12 | 7.33 | 4.26 | 7.14 | 10.52 | 9.90 | 2.43 | 6.90 |  |

**Table S2** Optimal substrate content, solvent and catalyst properties, and operational condition ranges detected *via* the MOPSO method for producing monomeric lignin.

| *Optimal inputs* | | | | | | | | | | | | | | | | *Optimal outputs* | | | | | | | |
| --- | --- | --- | --- | --- | --- | --- | --- | --- | --- | --- | --- | --- | --- | --- | --- | --- | --- | --- | --- | --- | --- | --- | --- |
| Substrate content | | | | | | Solvent and catalyst properties | | | | Operational conditions | | | | | | Monomer yields | | | | | | | |
| Cellulose  content (%) | | Hemicellulose content (%) | Lignin content (%) | | β-O-4  (%) | Polarity (RPS) | Solvent  RED | Metal: carbon (wt%) | Catalyst loading (wt%) | Time  (h) | Temperature (°C) | Total pressure (MPa) | Biomass loading (g/L) | Rotational speed (rpm) | H-  monomer | | S-  monomer | | G-  monomer | | Total  monomer | |  |
| 29.9 \| 55.7 | | 15.5 \| 27.1 | 13.1 \| 29.9 | | 59.7 \| 67.8 | 1.0 | 0.5 \| 0.6 | 1.0 \| 15.0 | 1.5 \| 5.4 | 2.0 \| 20.0 | 156 \| 287.7 | 4.1 \| 5.8 | 30.1 \| 47.2 | 385.5 \| 830.1 | 5.1 \| 9.9 | | 23.2 \| 34.5 | | 14.3 \| 15.5 | | 43.2 \| 59.0 | |  |
| *Randomly selected optimal conditions for experimental validation* | | | | | | | | | | | | | | | |  | |  | |  | |  | |
| **A** | 42.8 | 26.4 | | 20.4 | 62.4 | 1.0 | 0.5 | 5.0 | 2.1 | 2.6 | 170.3 | 4.5 | 46.9 | 582.2 | 8.6 | | 28.9 | | 14.6 | | 52.1 | |  |
| **B** | 45.6 | 19.9 | | 19.8 | 60.4 | 1.0 | 0.5 | 12.0 | 2.3 | 3.8 | 156.9 | 5.2 | 47.1 | 564.7 | 5.2 | | 23.2 | | 14.8 | | 43.2 | |  |
| **C** | 44.3 | 27.1 | | 20.5 | 60.7 | 1.0 | 0.5 | 7.0 | 3.5 | 2.8 | 160.3 | 4.5 | 46.9 | 586 | 5.1 | | 26.9 | | 14.6 | | 46.6 | |  |

*Based on randomly selected optimal conditions for experimental validation the following conditions were performed for conditions A, B, and C:*

A: Substrate (Aspen: 44% cellulose, 28% hemicellulose, 21% lignin, 66.6% β-O-4); Solvent (100 mL: 50 mL Water + 50 mL Ethylene glycol); Catalyst (Ru/C; 5 wt% Metal: carbon).

B: Substrate (Pine: 43.9% cellulose, 20.7% hemicellulose, 19.2% lignin, 58.7% β-O-4); Solvent (100 mL: 100 mL Ethylene glycol); Catalyst (Ru/C; 10 wt% Metal: carbon).

C: Substrate (Aspen: 44% cellulose, 28% hemicellulose, 21% lignin, 66.6% β-O-4); Solvent (100 mL: 50 mL Water + 50 mL Butanol); Catalyst (Ru/C; 5 wt% Metal: carbon).

**Table S3** Modules used in extracting data from Ecoinvent 3

| Inputs from Technosphere: energy/materials/transportation | Module on Simapro |
| --- | --- |
| Electricity | Electricity, high voltage {CN}\| market group for electricity, high voltage \| allocation, S |
| Heat | Heat, district or industrial, natural gas {RoW}\| market group for heat, district or industrial gas \| allocation, S  Heat, district or industrial, other than natural gas {GLO}\| heat production, coal tar, at industrial furnace- 1MW \| allocation, S |
| Diesel | Diesel {RoW}\| market for diesel |
| Transportation | Transport, truck 10-20t, Euro 5, 100%LF, empty return/GLO Mass |
| Dichloromethane | Dichloromethane {RoW}\| market for dichloromethane\| Conseq, U |
| Ethylene glycol | Ethylene glycol {GLO}\| market for \| Conseq, U |
| Pulp | Pulp, kraft market, bleached, average production, at mill/kg/RNA |
| Tap water | Tap water {GLO}\| market for \| Conseq, U |
| Pulp | Pulp, kraft market, bleached, average production, at mill/kg/RNA |
| Phenol | Phenol {RoW}\| market for phenol \| Conseq, U |

**Table S4** CAPEX for capacity of 660 kt/y in (M$) ^[1-7]^.

| CAPEX | | | | | | |
| --- | --- | --- | --- | --- | --- | --- |
| Process unit | | | | | | Cost |
| RCF area (reactor only costs) | | | | | | 122.7 |
| RCF area (Non-reactor costs) | | | | | | 40.1 |
| Storage | | | | | | 2.1 |
| Utilities | | | | | | 5.2 |
| Total installed equipment costs | | | | | | 170.2 |
| Direct + Indirect costs (% 46 of CAPEX) | | | | | | 145.0 |
| Total | | | | | | 315.2 |
| OPEX | | | | | |  |
| Items | | Cost in $/ton | | | M$/y (660 kt/y) | |
| Material cost | | | | | | |
| Aspen (wood) | | 95.6 | | | 63.1 | |
| Dichloromethane | | 350 | | | 2.9 | |
| H_2_ | | 3115 | | | 8.3 | |
| Ethylene glycol | | 660.8 | | | 25.3 | |
| Catalyst | | 418000 | | | 2.5 | |
| Electricity & heat | |  | | | 59.4 | |
| Operating labor | |  | | | 35.2 | |
| Maintenance | |  | | | 8.5 | |
| Fixed charge | |  | | | 29.3 | |
| Plant overhead cost | |  | | | 14.7 | |
| General expense | |  | | | 44.0 | |
| Total | |  | | | 293.3 | |
| Annual revenues | |  | | |  | |
| End-products | Cost in $/ton | | Product yield (ton/ton biomass) | M$/year | | |
| Pulp | 600 | | 431.4 | 170.8 | | |
| Lignin monomers | 1750 | | 88.9 | 102.7 | | |
| Xylose | 800 | | 233.6 | 123.3 | | |
| Total |  | |  | 396.9 | | |

**References**

[1] A. W. Bartling, M. L. Stone, R. J. Hanes, A. Bhatt, Y. Zhang, M. J. Biddy, R. Davis, J. S. Kruger, N. E. Thornburg, J. S. Luterbacher, R. Rinaldi, J. S. M. Samec, B. F. Sels, Y. Román-Leshkov, G. T. Beckham, *Energy Environ. Sci.* **2021**, *14*, 4147–4168.

[2] M. Tschulkow, T. Compernolle, S. Van den Bosch, J. Van Aelst, I. Storms, M. Van Dael, G. Van den Bossche, B. Sels, S. Van Passel, *J. Clean. Prod.* **2020**, *266*, DOI 10.1016/j.jclepro.2020.122022.

[3] Y. Liao, S. F. Koelewijn, G. van den Bossche, J. van Aelst, S. van den Bosch, T. Renders, K. Navare, T. Nicolaï, K. van Aelst, M. Maesen, H. Matsushima, J. M. Thevelein, K. van Acker, B. Lagrain, D. Verboekend, B. F. Sels, *Science (80-. ).* **2020**, *367*, 1385–1390.

[4] *SunSirs--China Commodity Data Group*. Available from: <https://www.sunsirs.com>.

[5] *Shanghai Metals Market (SMM)*. Available from: <https://price.metal.com/Hydrogen-Energy>.

[6] Available from: <https://www.made-in-china.com/>.

[7] Available from: <https://www.fastmarkets.com/>.
